# Occupational stress, work-life balance, and coping among female veterinarians in Germany

**DOI:** 10.3389/fvets.2026.1936424

**Published:** 2026-09-18

**Authors:** Lea Kolsch, László Ózsvári, Marietta Máté

**Affiliations:** Department of Veterinary Forensics and Economics, University of Veterinary Medicine Budapest, Budapest, Hungary

**Keywords:** coping strategies, emotional awareness, female veterinarians, occupational stress, resilience, veterinary mental health, work-life balance

## Abstract

**Introduction:**

Veterinary medicine involves substantial emotional and ethical responsibilities that may contribute to psychological strain. Work-related stressors, including long working hours, euthanasia, client-related challenges, and moral conflicts, have been associated with burnout and reduced mental well-being. This exploratory study examined occupational stress, work-life balance, and coping strategies among female veterinarians in Germany in relation to demographic, professional, and workplace characteristics.

**Methods:**

Given the gender composition of the veterinary workforce in Germany, with women representing the majority of veterinarians, this study focused on female veterinarians as a particularly relevant subgroup. An online questionnaire was distributed to veterinarians in Germany between September 2024 and September 2025. In total, 152 valid responses were analyzed using descriptive statistics, Pearson’s chi-square tests, Mann–Whitney U tests, and Kruskal–Wallis tests.

**Results:**

Working more than 40 h per week was associated with lower perceived workload manageability (*p* < 0.0001), less frequent conscious breaks, and more pronounced stress or burnout symptoms. Work-life balance showed the most consistent associations with stress- and recovery-related indicators: respondents with poor or very poor work-life balance reported lower workload manageability, less frequent conscious breaks, and more pronounced stress or burnout symptoms (all *p* < 0.0001). Respondents most frequently relied on informal and adaptive coping strategies, particularly talking to friends or family (84.2%), hobbies or leisure activities (69.7%), and physical exercise (57.9%), whereas professional counseling or therapy was reported by 15.1%. Avoidance or ignoring stress was more common among respondents with poor or very poor work-life balance (χ2 = 7.433, *p* < 0.05). Satisfaction with support for professional development and continuing education was associated with higher workload manageability, less pronounced stress or burnout symptoms, and more favorable ratings of team-based process optimization.

**Conclusion:**

Overall, the findings suggest that occupational stress and coping patterns among female veterinarians are associated with working hours, work-life balance, professional experience, and support for professional development and continuing education. These results highlight the importance of systemic approaches, including manageable working hours, protected breaks, collegial support, mentoring, improved workplace communication, and accessible psychological support, for promoting sustainable mental health in veterinary medicine.

## 1 Introduction

### 1.1 Occupational stress and mental health in veterinary medicine

Veterinary medicine is a highly demanding profession that requires not only advanced professional knowledge and clinical decision-making skills, but also substantial psychological resilience. Veterinarians are regularly exposed to ethically, emotionally, and economically challenging situations, including animal suffering, euthanasia, communication difficulties with animal owners, financial constraints affecting treatment decisions, long working hours, night and weekend duties, and emergency care (1–10). Although the veterinary profession is often perceived as socially respected and is sometimes idealized by the public, the psychological burden associated with everyday veterinary work is frequently underestimated (1–3).

A growing body of international research has shown that veterinarians are at increased risk of work-related stress, burnout, psychological distress, depression, and suicidal ideation (7–13). High workload and chronic occupational stress may contribute to burnout and other mental health problems, particularly when recovery opportunities and access to social or organizational support are insufficient. In the United States, veterinarians have been reported to show higher levels of burnout and suicidal behavior than the general population (11). Similar concerns have also been described in Europe. In Germany, veterinarians were found to have an approximately sevenfold higher risk of developing depression compared with the general population, and 19.2% of participating veterinarians reported suicidal thoughts within the previous two weeks (12).

### 1.2 Coping, emotional awareness, resilience, and social support

Coping strategies play a central role in determining whether work-related stressors are followed by adaptive adjustment or persistent psychological strain. Coping is commonly distinguished into problem-focused and emotion-focused strategies, depending on whether the individual attempts to address the stressor itself or to regulate the emotional response associated with it (14). Adaptive coping strategies, such as active problem-solving, seeking social support, positive reframing, acceptance, humor, relaxation techniques, and structured self-care, may help reduce stress and support long-term mental well-being (14, 15). By contrast, maladaptive strategies, including avoidance, rumination, self-blame, resignation, emotional withdrawal, overworking, alcohol use, or other forms of self-medication, may provide short-term relief while increasing the risk of burnout, compassion fatigue, depression, and other long-term psychological consequences (1, 8, 14, 16, 17). The usefulness of a coping strategy may also depend on the controllability of the situation: problem-focused approaches may be more appropriate when the stressor can be modified, whereas emotion-focused approaches may be more helpful when the situation cannot be changed, as is often the case in veterinary practice (14, 15, 18).

Emotional awareness and emotion regulation are therefore important components of stress management in veterinary work. In the veterinary literature, overlapping terms such as emotional competence, emotional intelligence, emotional awareness, self-awareness, empathy, and resilience are used to describe the ability to recognize, understand, regulate, and constructively use one’s own and others’ emotions (19–22). These capacities may help veterinarians remain professionally effective in emotionally demanding situations while also supporting long-term mental well-being (19, 20). Empathy is particularly relevant in veterinary medicine, as it enables veterinarians to respond sensitively to both animal suffering and owners’ distress. At the same time, high empathic involvement may become a source of vulnerability when emotional boundaries are insufficiently developed, potentially contributing to compassion fatigue (19, 21). Personality traits such as perfectionism, conscientiousness, and fear of failure may further increase vulnerability to anxiety, psychological strain, burnout, and moral stress, especially when veterinarians set rigidly high standards for themselves or experience guilt when optimal care cannot be provided (22, 23).

Critical incidents in veterinary practice, such as unexpected complications, the death of an animal, euthanasia, adverse outcomes, or treatment limitations caused by owners’ financial constraints, may evoke guilt, grief, anxiety, self-doubt, helplessness, frustration, and even physiological symptoms of stress (3, 6, 16, 24). In such situations, veterinarians may rely on mental compartmentalization, distraction, emotional distancing, physical activity, or alcohol use to continue working and reduce acute emotional tension (16). While some distancing strategies may be functional in the short term, persistent emotional detachment may become problematic if it leads to reduced empathy, impaired communication, or lower quality of care (25). Moral distress is particularly relevant in this context, as it arises when veterinarians feel unable to act in accordance with their professional or ethical values due to external constraints, such as economic limitations, owner expectations, or institutional barriers (3, 17, 22, 23). Repeated exposure to such conflicts may contribute to guilt, helplessness, anger, emotional numbness, compassion fatigue, burnout, and, in severe cases, suicidal ideation (3, 16, 23, 26).

The consequences of stress and ineffective coping are not limited to veterinarians’ personal well-being. Psychological strain may also affect professional performance, concentration, decision-making, communication with clients and colleagues, team dynamics, and patient safety (3, 27, 28). High stress levels may increase susceptibility to errors or reduce the ability to process important clinical information, while unresolved moral distress may lead to frustration, disengagement, and impaired decision-making (3, 29, 30). Therefore, supporting veterinarians’ mental well-being is relevant not only from an occupational health perspective, but also to maintaining the quality and sustainability of veterinary care (29, 30).

Resilience in veterinary medicine should not be understood as a fixed personal trait, but rather as a dynamic process shaped by professional experience, reflection, self-efficacy, social support, and organizational context (2, 15, 16, 18, 19). Constructive processing of critical events may include technical learning, broader perspective-taking, collegial discussion, and the development of personal emotion-regulation strategies (16). Higher self-efficacy, the use of personal resources, and adaptive emotion-focused coping have been associated with greater resilience, whereas self-blame and avoidance appear to be negatively related to resilience (18). Career stage is also relevant: students and new graduates often experience high performance pressure, reality shock, uncertainty, and limited preparation for the emotional demands of practice, which may increase stress and the use of ineffective coping strategies (31–35). Research among Hungarian veterinary students has also shown that career choice in veterinary medicine is strongly shaped by motivational factors before entering the profession, which may be relevant to later expectations, career commitment, and professional identity (36). In contrast, more experienced veterinarians may report greater composure, clearer priorities, lower burnout and secondary traumatic stress, and higher compassion satisfaction, although cumulative exposure to ethical conflicts and long-term workload may remain burdensome (2, 19, 37, 38).

Social support is one of the most consistently identified protective factors in veterinary mental health. Supportive relationships at work and in private life can provide emotional relief, practical assistance, professional learning, and a sense of shared experience (18–20, 39). Collegial support after critical incidents may reduce self-doubt, enable technical reflection, and facilitate emotional processing, whereas insensitive criticism or lack of recognition may increase stress (9, 39). Supportive work environments, open communication, mentoring, structured feedback, supervision, and an open error culture may therefore act as important organizational resources (19, 23, 39). Private relationships are also essential, as family, friends, and pets may provide emotional stability, recovery, and a source of identity outside the workplace (18, 21, 40). However, long working hours and insufficient leisure time can impair work-life balance by reducing opportunities for social connection and recovery, thereby increasing isolation and psychological strain (23).

### 1.3 Gender, work-life balance, and occupational context

Gender is another important factor in the experience and management of occupational stress in veterinary medicine. Female veterinarians, particularly those in early career stages, have been reported to experience higher levels of stress, anxiety, depression, and psychological vulnerability than their male counterparts (7, 8, 11, 23, 33, 37). Women may be more likely to seek interpersonal support and professional counseling, and they often place greater emphasis on a positive working atmosphere, flexible working conditions, and family-friendly work organization (7, 20, 23, 27, 28). Nevertheless, studies focusing on female veterinarians indicate that maladaptive coping strategies, such as rumination, resignation, and self-blame, may also be common in this group (8). Considering the ongoing feminization of the veterinary profession, especially among students and young professionals, a better understanding of occupational stress, work-life balance, and coping among female veterinarians is particularly important for the development of targeted prevention and support strategies (8, 19).

Although occupational stressors, burnout, suicidality, and maladaptive coping have been widely discussed in the veterinary literature, research remains partly deficit-oriented, with comparatively less emphasis on protective resources, meaningfulness, compassion satisfaction, job satisfaction, and successful coping (17, 19, 26). Positive aspects of veterinary work, including meaningful animal care, successful treatments, gratitude from owners, collegial recognition, professional growth, and compassion satisfaction, may buffer against burnout and support long-term well-being (17, 18, 26, 39, 41, 42). Nevertheless, there is still limited knowledge about how veterinarians, particularly female veterinarians, experience occupational stress, maintain work-life balance, and use coping strategies in response to professional demands.

Therefore, this exploratory study aimed to examine occupational stress, work-life balance, and coping among veterinarians in Germany in relation to demographic, professional, and workplace characteristics. Given the very small number of male respondents, the analytical sample was restricted to female veterinarians. Specifically, it sought to identify adaptive and maladaptive coping strategies and to assess their associations with age, marital status, weekly working hours, professional position and experience, on-call duties, work-life balance, and perceived support for professional development and continuing education.

## 2 Materials and methods

### 2.1 Survey methodology and questionnaire

The survey was conducted using an online questionnaire. An extensive literature review of coping strategies used in veterinary medicine and their potential consequences (7, 14, 19, 26, 32, 40, 42) formed the basis for the initial draft of the questionnaire. Several rounds of revision were undertaken to ensure that the questionnaire covered a broad range of occupational stressors, emotional responses, and coping strategies relevant to veterinary practice, while also enabling the identification of potential areas for improvement. The content of the questionnaire was organized around domains that had been repeatedly identified in the veterinary mental health and coping literature, including occupational stress, emotional awareness, emotion regulation, social support, workload, recovery, adverse events, euthanasia, client-related challenges, and workplace support. The specifically worded Likert-scale items were used to capture comparable self-assessments of these domains across respondents, while the situation-specific questions were intended to reflect common emotionally demanding events in veterinary practice. Predominantly closed-ended and semi-closed response formats were chosen to allow standardized quantitative comparisons across demographic, professional, and workplace-related groups. To retain an exploratory element, semi-closed response options and an open-ended question were included so that participants could report additional strategies, experiences, or support needs not covered by the predefined categories.

The original questionnaire was developed in English, and the finalized version (Supplementary material Questionnaire) was translated into German by a native German-speaking co-author. The translation was reviewed collaboratively by the research team, and discrepancies in meaning or phrasing were resolved by consensus to ensure conceptual and linguistic equivalence between the English and German versions, therefore, no back-translation was performed. Before the survey was launched, a pilot test was conducted with veterinary practitioners (*n* = 9) to ensure that all items were clearly understandable and that no ambiguities or technical issues remained. No formal psychometric validation of the questionnaire was performed; the pilot testing was intended to improve clarity, comprehensibility, and practical applicability. Feedback from the pilot test was used to refine the wording and improve overall clarity.

Participants received a link to the questionnaire along with a short description outlining the survey’s objectives, the estimated completion time of approximately 15 min, and assurances of anonymity. Participation was voluntary and anonymous, and all participants provided informed consent electronically before completing the survey. Recruitment followed a convenience sampling approach through social media channels, veterinary mailing lists, and veterinary newsletters. Due to the exploratory nature of the study, no *a priori* sample size calculation was conducted; instead, the aim was to obtain as large and diverse a sample as possible during the survey period. This recruitment strategy was chosen to reach veterinarians from different geographical regions across Germany.

Eligible participants were required to hold a veterinary degree and to be currently working in any field of the veterinary profession. Recruitment was not restricted by gender. Incomplete or invalid questionnaires were excluded from the analysis. Only five male veterinarians completed the questionnaire, and this subgroup was considered too small for a meaningful separate analysis. Their responses were therefore excluded from the analytical sample. After excluding the five male respondents and two incomplete questionnaires, the final analytical sample comprised 152 female veterinarians. Before analysis, the dataset was checked for completeness, logical consistency, and potential duplicate responses. As the questionnaire was anonymous, individual identification was not possible; however, responses were manually reviewed for implausible or internally inconsistent combinations of demographic and employment-related variables. The questionnaire was accessible via Google Forms from 12 September 2024 to 19 September 2025, and the data were exported to Microsoft Excel for data preparation before statistical analysis in IBM SPSS Statistics.

The survey comprised 50 questions, including 40 closed-ended questions, 9 semi-closed questions, and 1 open-ended question. Questions 1–11 collected sociodemographic and occupational information such as age, gender, marital status, federal state, size of place of residence, professional field, average weekly working hours, employment status, years of professional experience, on-call duties, and self-rated general health. Questions 12–24 included statements rated on a five-point Likert scale and assessed the work environment, emotional awareness, and stress-management skills of the participants. Response options ranged from “strongly disagree” to “strongly agree.” Additional categorical items were also included in this section. Questions 25–35 examined general stress-management and coping strategies, while Questions 36–47 explored emotional and behavioral responses to specific workplace situations. These included emotional reactions to mistakes, preparation for euthanasia, responses to client complaints, and the management of aggressive animals. Depending on the item, participants responded using five-point Likert scales, single-choice questions, or multiple-choice questions were used. The final section (Questions 48–50) asked participants to reflect on potential improvements and to identify resources, organizational changes, or training opportunities that could support stress management and emotional well-being; it also provided space for additional comments.

### 2.2 Statistical analysis

The data were analyzed using IBM SPSS Statistics Version 25 (IBM Corp., Armonk, NY, USA) (43). The individual veterinarian was the unit of analysis. Descriptive statistics were first calculated to summarize the characteristics of the sample and the distribution of responses. Categorical variables were presented as frequencies and percentages. For items measured on a five-point Likert scale, means and standard deviations (mean ± SD) were calculated to summarize response patterns and facilitate interpretation, although the items were treated as ordinal variables in the inferential analyses.

The Likert-scale items assessed emotional awareness, stress-management skills, perceived workload, collegial and client-related support, and emotional and professional responses to stressful workplace situations. The response options ranged from 1 = strongly disagree to 5 = strongly agree. The distributions of the Likert-scale variables were assessed using the Shapiro–Wilk test. As all Likert-scale variables deviated significantly from normality (*p* < 0.05) and were ordinal in nature, non-parametric tests were applied for group comparisons.

For comparisons between two independent groups, such as groups based on marital status, weekly working hours, and professional position, the Mann–Whitney U test was used. Results were reported with the U statistic, Z value, and two-tailed *p*-value. For comparisons involving three or more independent groups, such as age group, professional experience, frequency of on-call shifts, perceived work-life balance, and satisfaction with support for professional development and continuing education, the Kruskal–Wallis test was applied. Results were reported with the H, degrees of freedom (df) and *p*-value. Where a Kruskal–Wallis test indicated a statistically significant difference, the group-level patterns were interpreted using the corresponding descriptive statistics.

Pearson’s chi-square (χ^2^) tests were used to examine associations between categorical variables. These analyses assessed whether demographic and occupational characteristics, including age group, marital status, weekly working hours, professional position and experience, frequency of on-call shifts, perceived work-life balance, and satisfaction with support for professional development and continuing education, were associated with categorical questionnaire responses. Multiple-response questions were coded as separate binary variables for each response option, indicating whether each option had or had not been selected by the respondent. The assumptions of the chi-square test, including expected cell frequencies, were checked before interpretation. Chi-square results were reported with the χ^2^ statistic, degrees of freedom (df) and *p*-value. Effect sizes for chi-square analyses were calculated using Cramer’s V where appropriate. Associations were interpreted with reference to conventional thresholds (44), with Cramer’s V values of approximately 0.10, 0.30, and 0.50 generally indicating small, medium, and large effects, respectively. Given the exploratory nature of the study and the use of convenience sampling, all inferential results were interpreted cautiously. A significance level of *p* < 0.05 was applied to all statistical analyses. Given the large number of exploratory comparisons, *p*-values from the inferential analyses were additionally adjusted using the Benjamini–Hochberg false discovery rate (FDR) procedure. Both unadjusted and FDR-adjusted *p*-values were considered when interpreting the findings. Results that remained significant after FDR adjustment were considered more robust, whereas those that were significant only before adjustment were interpreted as exploratory patterns. Given the exploratory nature of the study and the use of convenience sampling, all inferential analyses were interpreted cautiously and regarded as hypothesis-generating.

## 3 Results

### 3.1 Socio-demographic and occupational characteristics of the sample

The final sample comprised 152 female veterinarians, whose socio-demographic characteristics are presented in Table 1. The largest age group comprised respondents aged 23 to 34 years (66/152; 43.4%), and most worked in small animal practice (108/152; 71.1%). For comparison, the 2024 statistics of the German Federal Chamber of Veterinarians reported 6,241 small-animal practitioners among 11,264 self-employed veterinarians in practice, corresponding to approximately 55.4%. Among female self-employed veterinarians in practice, 4,354 of 6,569 worked in small animal practice, corresponding to approximately 66.3% (45). Thus, small animal practitioners were overrepresented in the present sample. However, because directly comparable national statistics by field of practice are limited for employed veterinarians, and because official age categories do not fully correspond to the age groups used in the present study, no formal comparison with the full target population was performed. Therefore, the findings should be interpreted as exploratory.

**Table 1:** Socio-demographic and occupational characteristics of the respondents (n = 152).

Variable	Category	Number of responses (*n* = 152)	Percentage of responses (%)
Age	23–34 years	66	43.4%
	35–44 years	56	36.8%
	> 45 years	30	19.7%
Marital status	Unmarried	96	63.2%
	Married	56	36.8%
Size of place of residence	City (> 100.000)	39	25.7%
	Middle town (20.001–100.000)	31	20.4%
	Small town (5.000–20.000)	38	25.0%
	Rural community (< 5.000)	44	28.9%
Field of veterinary work	Small animal veterinarian	108	71.1%
	Equine veterinarian	26	17.1%
	Farm animal veterinarian	21	13.8%
	Exotic animal veterinarian	3	2.0%
	Research/education	3	2.0%
	Authorities/state veterinarian	2	1.3%
	Pharmaceutical industry	1	0.7%
	Laboratory diagnostic	1	0.7%
	Other	7	4.6%
Weekly working hours	≤ 40 h	95	62.5%
	> 40 h	57	37.5%
Employment status	Employed	120	78.9%
	Employer	32	21.1%
Professional experience	0–5 years	66	43.4%
	6–10 years	32	21.1%
	11–20 years	39	25.7%
	> 20 years	15	9.9%
Frequency of on-call shifts	No	48	31.6%
	Yes, cc. once a month	34	22.4%
	Yes, 2-3x per month	20	13.2%
	Yes, once a week	19	12.5%
	Yes, more than once a week	31	20.4%
Work-life balance	Excellent or Good	51	33.6%
	Fair	67	44.1%
	Poor or very poor	34	22.4%
Satisfaction with support for professional development and continuing education	Very satisfied or satisfied	77	50.7%
	Neutral	33	21.7%
	Dissatisfied or very dissatisfied	42	27.6%

Field of veterinary work: As multiple responses were allowed, percentages may total more than 100%.

Responses in the “Other” category included specialized veterinary roles such as New World camelid medicine (e.g., llamas and alpacas), veterinary medical IT and software development, military veterinary service, university-based pathology, and animal shelter practice.

Participants were distributed across most German federal states, with the largest proportions working in Lower Saxony (34/152; 22.4%), North Rhine-Westphalia (32/152; 21.1%), and Bavaria (28/152; 18.4%). Regarding work-life balance, only 51/152 participants (33.6%) rated it as excellent or good, while the largest proportion described it as fair (67/152; 44.1%). Respondents reporting poor work-life balance most frequently rated their general health status as poor (29/34; 85.3%), whereas those reporting good work-life balance mainly rated their health as moderate to good (38/51; 74.5%) (χ^2^ = 33.023, *p* < 0.0001).

### 3.2 Emotional awareness, stress-management, and general coping strategies

#### 3.2.1 Emotional awareness, workload, and recovery indicators

Participants reported relatively high emotional awareness, as reflected by their agreement with the statement that they were aware of their feelings and emotions as they occurred (mean: 4.02 ± 0.78) (Figure 1). They also reported strong personal relationships with partners, friends, family members, or colleagues (mean: 4.41 ± 0.89). In contrast, lower mean scores were observed for emotion regulation and boundary setting in challenging situations (mean: 2.95 ± 0.95), consciously taking breaks without dwelling on work-related stressors (mean: 2.95 ± 1.21), and perceived workload manageability (mean: 2.93 ± 1.08) (Figure 2).

**Figure 1:**
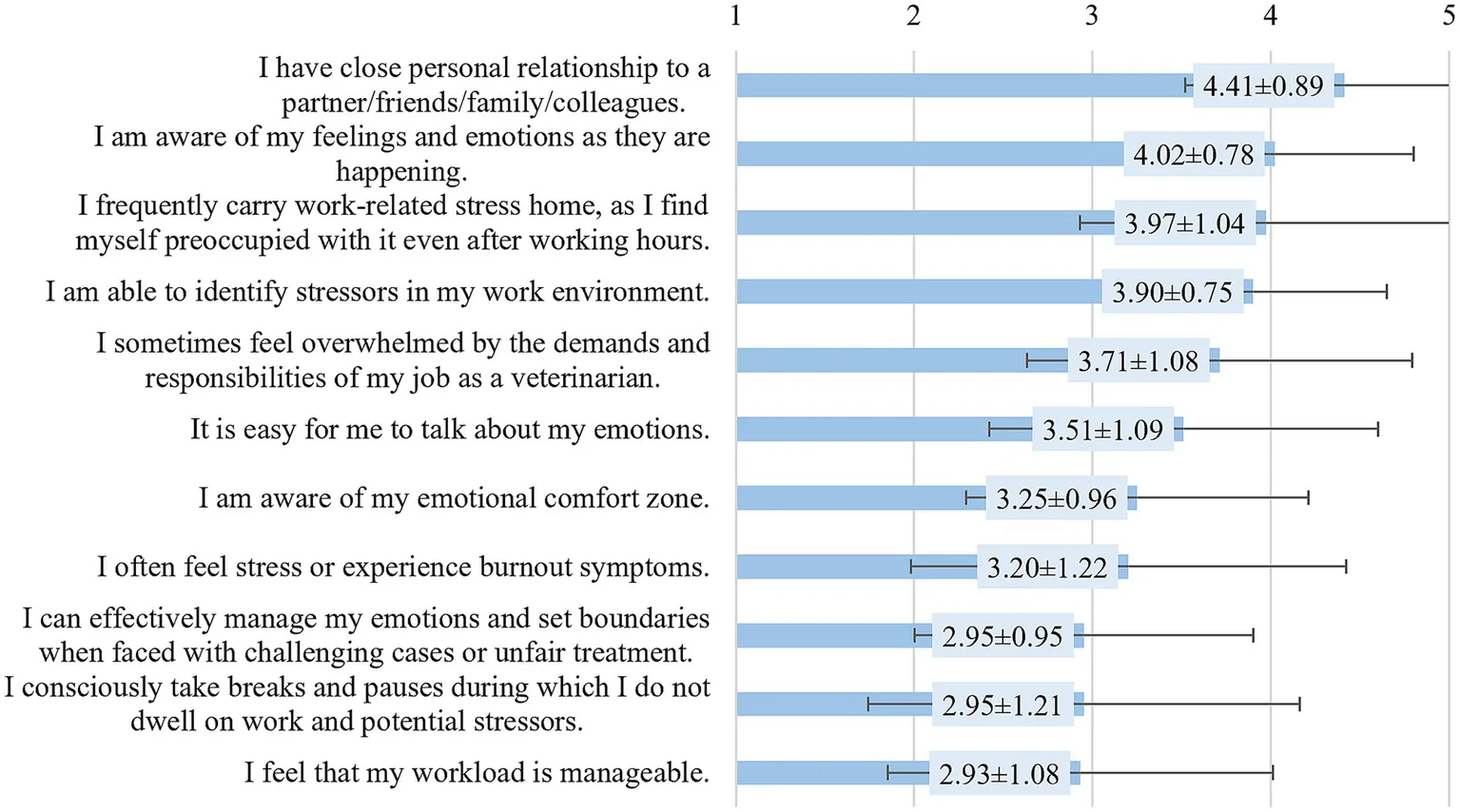
Female veterinarians’ perceptions of their emotional awareness and stress-management skills (mean ± SD; *n* = 152). Responses were given on a five-point Likert scale, where 1 = strongly disagree, 2 = disagree, 3 = neither agree nor disagree, 4 = agree, and 5 = strongly agree.

**Figure 2:**
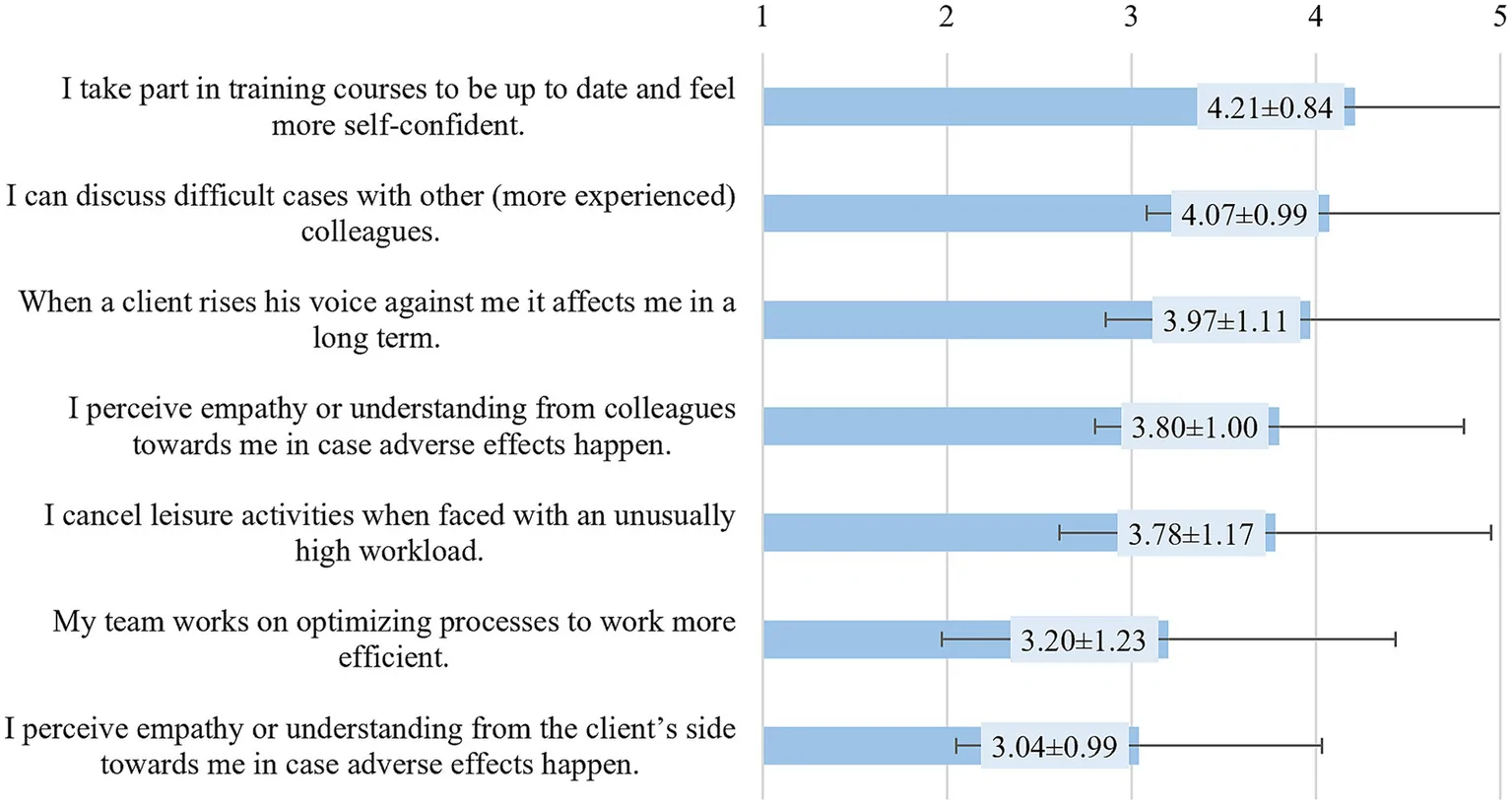
Female veterinarians’ responses to workload, client interactions, collegial support, and professional confidence (mean ± SD; *n* = 152). Responses were given on a five-point Likert scale, where 1 = strongly disagree, 2 = disagree, 3 = neither agree nor disagree, 4 = agree, and 5 = strongly agree.

#### 3.2.2 Workload, work-life balance, and stress-related indicators

Supplementary Tables 1–8 provide detailed results by socio-demographic and occupational variables. Age showed descriptive patterns related to emotional strain: veterinarians aged 23–34 years reported the highest mean score for feeling overwhelmed (mean: 3.94 ± 1.04), followed by those older than 45 years (mean: 3.67 ± 0.88) and those aged 35–44 years (mean: 3.46 ± 1.18). Stress or burnout symptoms showed a similar pattern, with the highest mean score among veterinarians aged 23–34 years (mean: 3.45 ± 1.15), followed by those older than 45 years (mean: 3.23 ± 1.19) and those aged 35–44 years (mean: 2.88 ± 1.25). Professional experience remained significantly associated with feeling overwhelmed, with the highest mean score among veterinarians with 0–5 years of experience (mean: 4.02 ± 1.03) and the lowest among those with more than 20 years of experience (mean: 3.33 ± 0.62) (Kruskal–Wallis test: H (3) = 13.683, *FDR-adjusted p* < 0.05).

Workload-related factors showed several patterns related to stress management and recovery, with the strongest evidence observed for lower workload manageability among veterinarians working more than 40 h per week. Veterinarians working more than 40 h per week reported taking conscious breaks less often (mean: 2.63 ± 1.19) than those working 40 h or less (mean: 3.15 ± 1.19). They also reported lower workload manageability (mean: 2.51 ± 0.98 vs. 3.19 ± 1.07; Mann–Whitney U test: U = 1717.0, Z = −3.909, *FDR-adjusted p* < 0.001) and higher scores for stress or burnout symptoms (mean: 3.46 ± 1.24 vs. 3.04 ± 1.18). On-call duties were also associated with emotion regulation and boundary setting: veterinarians without on-call duties reported the highest agreement with being able to regulate emotions and set boundaries in challenging situations (mean: 3.23 ± 0.97), whereas the lowest agreement was observed among those with more than one on-call shift per week (mean: 2.58 ± 0.92).

Professional position showed descriptive differences in several indicators of emotion regulation and recovery. Employers reported higher scores for emotion regulation and boundary setting (mean: 3.25 ± 0.95) than employed veterinarians (mean: 2.88 ± 0.94). Employers also reported taking conscious breaks more often (mean: 3.38 ± 1.19 vs. 2.84 ± 1.20) and greater awareness of their emotional comfort zone (mean: 3.63 ± 0.98 vs. 3.15 ± 0.94).

Work-life balance showed the most consistent associations with stress- and recovery-related indicators. Mean scores for taking conscious breaks were highest among respondents with excellent or good work-life balance (mean: 3.63 ± 1.31) followed by those with fair (mean: 2.82 ± 1.15) and poor or very poor work-life balance (mean: 2.21 ± 0.91) (Kruskal–Wallis test: H (2) = 29.172, *FDR-adjusted p* < 0.0001). A similar pattern was observed for workload manageability, with mean scores of 3.76 ± 0.82 among respondents with excellent or good work-life balance, 2.81 ± 0.94 among those with fair work-life balance, and 1.94 ± 0.69 among those with poor or very poor work-life balance (Kruskal–Wallis test: H (2) = 61.417, *FDR-adjusted p* < 0.0001). In contrast, respondents with poor or very poor work-life balance reported the highest mean score for carrying work-related stress home (mean: 4.53 ± 0.66), followed by those with fair (mean: 3.84 ± 1.12) and excellent or good work-life balance (mean: 3.76 ± 1.03) (Kruskal–Wallis test: H (2) = 13.124, *FDR-adjusted p* < 0.05). They reported the highest mean score for feeling overwhelmed (mean: 4.09 ± 1.00), compared with respondents with excellent/good (mean: 3.67 ± 1.13) or fair work-life balance (mean: 3.55 ± 1.05). Similarly, stress or burnout symptom scores were highest among respondents with poor or very poor work-life balance (mean: 4.12 ± 1.07), followed by those with fair (mean: 3.22 ± 1.06) and excellent or good work-life balance (mean: 2.55 ± 1.12) (Kruskal–Wallis test: H (2) = 34.591, *FDR-adjusted p* < 0.0001).

Satisfaction with support for professional development and continuing education was also associated with more favorable stress-related indicators. Mean workload manageability scores were highest among veterinarians who were satisfied or very satisfied with such support (mean: 3.17 ± 1.08) followed by neutral respondents (mean: 2.94 ± 1.00) and those who were dissatisfied or very dissatisfied (mean: 2.50 ± 1.04) (Kruskal–Wallis test: H (2) = 10.846, *FDR-adjusted p* < 0.05). Stress or burnout symptom scores showed the opposite pattern, with mean values of 2.81 ± 1.15 among satisfied or very satisfied respondents, and 3.33 ± 1.05 among neutral respondents, and 3.81 ± 1.22 among dissatisfied or very dissatisfied respondents (Kruskal–Wallis test: H (2) = 19.755, *FDR-adjusted p* < 0.01). The mean score for carrying work-related stress home was also lowest among respondents who were satisfied or very satisfied with support for professional development and continuing education (mean: 3.73 ± 1.11).

#### 3.2.3 General coping strategies and break-taking behavior

Regarding general stress-management techniques, respondents most frequently reported using adaptive or supportive strategies, particularly talking to friends or family (128/152; 84.2%), engaging in hobbies or leisure activities (106/152; 69.7%), and participating in physical exercise, such as running, swimming, gym workouts, or yoga (88/152; 57.9%) (Table 2). In contrast, more structured forms of psychological support or reflective coping were less frequently reported: 29/152 respondents (19.1%) used meditation or mindfulness practices, and 23/152 respondents (15.1%) reported using professional counseling or therapy. Maladaptive or potentially less effective coping strategies were also reported, with 61/152 respondents (40.1%) avoiding or ignoring stress and 30/152 respondents (19.7%) reporting alcohol use as a means of managing stress.

**Table 2:** Stress-management techniques regularly used by female veterinarians (%; *n* = 152).

Stress-management techniques	Number of responses (*n*)	Percentage of responses (%)
Talking to friends or family	128	84.2%
Hobbies or leisure activities	106	69.7%
Physical exercise (e.g., running, swimming, gym workouts, yoga)	88	57.9%
Avoiding or ignoring stress	61	40.1%
Alcohol use	30	19.7%
Meditation or mindfulness practices	29	19.1%
Professional counseling or therapy	23	15.1%
Other	7	4.6%

As multiple responses were allowed, percentages may total more than 100%.

Responses in the “Other” category included sleeping, confronting difficult clients directly and discouraging them from returning to the practice, eating, yoga, and walking the dog.

Work-life balance was significantly associated with the use of several stress management techniques. Veterinarians with excellent or good work-life balance reported talking to friends or family more frequently (47/51; 92.2%) than those with fair (59/67; 88.1%) or poor or very poor work-life balance (22/34; 64.7%) (χ^2^ = 12.896, *FDR-adjusted p* < 0.05). Similarly, respondents with excellent or good work-life balance reported using hobbies or leisure activities more often (46/51; 90.2%) than those with fair (41/67; 61.2%) or poor or very poor work-life balance (19/34; 55.9%) (χ^2^ = 15.524, *FDR-adjusted p* < 0.01). In contrast, avoiding or ignoring stress was more common among veterinarians with poor or very poor work-life balance (20/34; 58.8%) than among those with fair (26/67; 38.8%) or excellent or good work-life balance (15/51; 29.4%) (χ^2^ = 7.433, *FDR-adjusted p* < 0.05). Weekly working hours were also associated with engagement in hobbies or leisure activities, which was reported more frequently by veterinarians working 40 h or less per week (72/95; 75.8%) than by those working more than 40 h per week (34/57; 59.6%).

Taking breaks during the workday varied among respondents. More than half reported taking breaks once or twice a day (86/152; 56.6%), while 46/152 respondents (30.3%) rarely took breaks and 6/152 respondents (3.9%) never did so. Only 14/152 respondents (9.2%) reported taking breaks every 2–3 h, and none of the respondents reported taking breaks hourly (0/152; 0.0%). No significant association was observed between age group and break-taking frequency.

### 3.3 Workplace support and responses to adverse events and mistakes

#### 3.3.1 Workplace support and team-related indicators

Participants reported the highest agreement with the statement that they participated in training courses to remain up to date and increase their self-confidence (mean: 4.21 ± 0.84), followed by the statement that they were able to discuss difficult cases with more experienced colleagues (mean: 4.07 ± 0.99) (Figure 3). In contrast, lower mean scores were observed for team-based process optimization (mean: 3.20 ± 1.23) and perceived empathy or understanding from clients following adverse events (mean: 3.04 ± 0.99).

**Figure 3:**
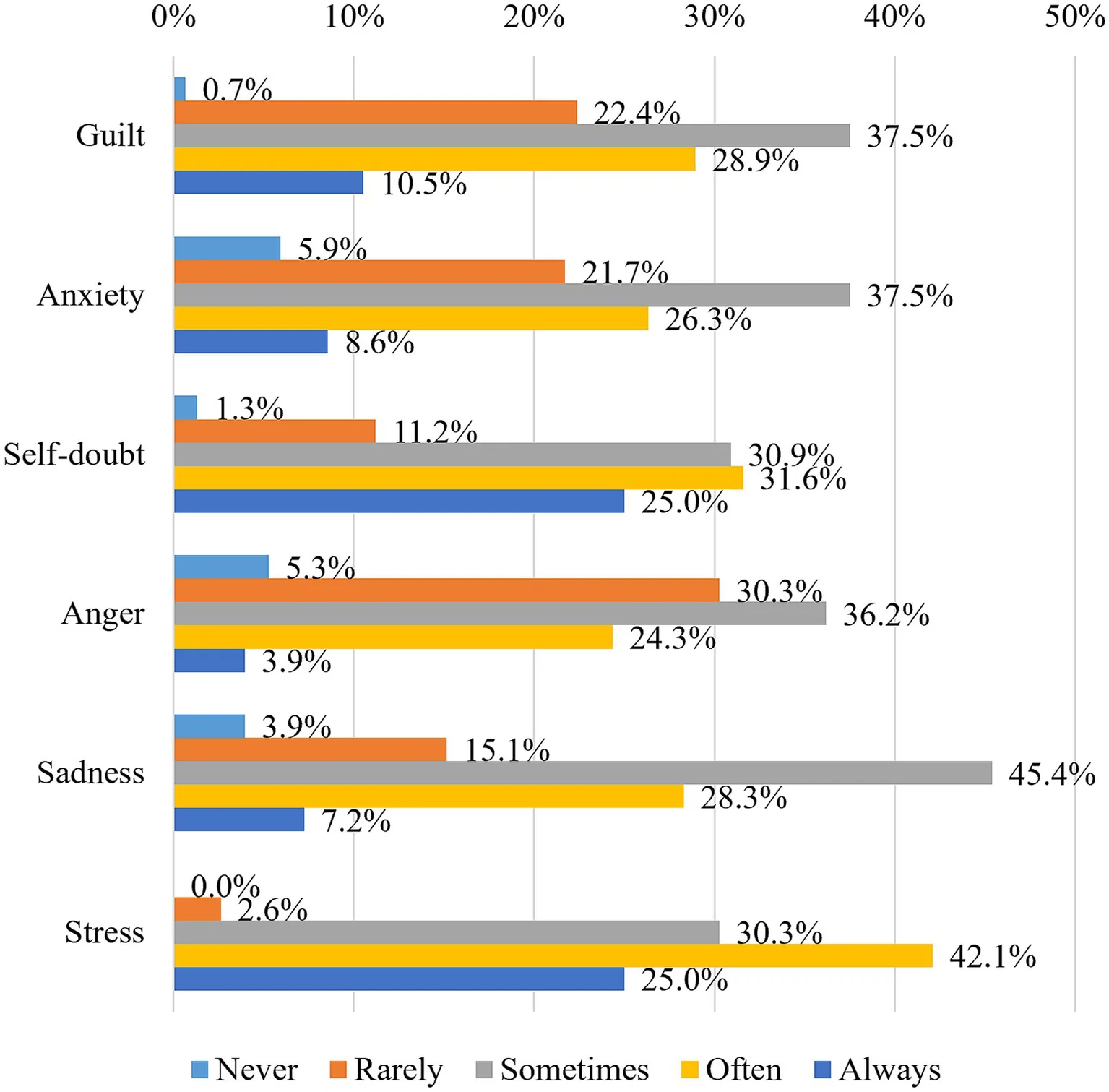
Frequency of emotional reactions reported by female veterinarians following adverse events (%; *n* = 152). Responses were given on a five-point frequency scale, where 1 = never, 2 = rarely, 3 = sometimes, 4 = often, and 5 = always. Percentages may not total exactly 100% because of rounding.

Supplementary Tables 9–16 provide detailed results by socio-demographic and occupational variables. Several demographic and professional characteristics were associated with organizational and client-related experiences. Agreement that the team worked to optimize processes and improve efficiency was highest among veterinarians older than 45 years (mean: 3.60 ± 1.40), followed by those aged 35–44 years (mean: 3.38 ± 1.15) and those aged 23–34 years (mean: 2.88 ± 1.14). Married respondents also reported higher scores for team-based process optimization (mean: 3.48 ± 1.21) than unmarried respondents (mean: 3.04 ± 1.22).

Professional position was associated with team-based process optimization and showed differences in other organizational and client-related indicators. Employers reported higher scores for team-based process optimization (mean: 3.88 ± 1.07) than employed veterinarians (mean: 3.03 ± 1.21) (Mann–Whitney U test: U = 1166.5, Z = −3.495, *FDR-adjusted p* < 0.01). Employers perceived greater empathy or understanding from clients following adverse events (mean: 3.50 ± 1.19) than employed veterinarians (mean: 2.92 ± 0.89). Professional experience was also associated with several responses. The ability to discuss difficult cases with more experienced colleagues differed by professional experience, with high scores among veterinarians with 0–5 years of experience (mean: 4.30 ± 0.82) and those with more than 20 years of experience (mean: 4.27 ± 1.10), while lower scores were observed among those with 6–10 years (mean: 3.88 ± 1.07) or 11–20 years of experience (mean: 3.74 ± 1.07). Veterinarians with more than 20 years of professional experience were more likely to agree that their team worked on optimizing processes and improving efficiency, whereas those with 0–5 years of experience showed the lowest agreement with this statement (mean: 3.80 ± 1.32 vs. 2.97 ± 1.11). Similarly, veterinarians with more than 20 years of experience reported the highest perceived empathy or understanding from clients following adverse events (mean: 3.80 ± 0.94), whereas lower agreement was observed in the other experience groups.

Work-life balance showed the most consistent associations with workload- and support-related responses. Respondents with poor or very poor work-life balance reported the highest mean score for canceling leisure activities when faced with an unusually high workload (mean: 4.24 ± 1.05), followed by those with fair (mean: 3.90 ± 1.16) and excellent or good work-life balance (mean: 3.31 ± 1.12) (Kruskal–Wallis test: H (2) = 17.572, *FDR-adjusted p* < 0.01). The mean score for the ability to discuss difficult cases with more experienced colleagues was highest among respondents with excellent or good work-life balance (mean: 4.39 ± 0.70), followed by those with fair (mean: 4.03 ± 0.94) and poor or very poor work-life balance (mean: 3.65 ± 1.30). Mean scores for team-based process optimization showed a similar pattern, with values of 3.53 ± 1.19 among respondents with excellent or good work-life balance, 3.28 ± 1.17 among those with fair work-life balance, and 2.56 ± 1.21 among those with poor or very poor work-life balance (Kruskal–Wallis test: H (2) = 12.653, *FDR-adjusted p* < 0.05).

Perceived empathy or understanding from clients and colleagues following adverse events also differed by work-life balance. Respondents with excellent or good work-life balance reported the highest perceived empathy or understanding from clients following adverse events (mean: 3.39 ± 0.87), followed by those with fair work-life balance (mean: 3.12 ± 0.90) and poor or very poor work-life balance (mean: 2.35 ± 1.01) (Kruskal–Wallis test: H (2) = 20.410, *FDR-adjusted p* < 0.01). A similar pattern was observed for perceived empathy or understanding from colleagues, with the highest agreement among respondents with excellent or good work-life balance (mean: 4.18 ± 0.77), followed by those with fair work-life balance (mean: 3.75 ± 0.99) and poor or very poor work-life balance (mean: 3.35 ± 1.15) (Kruskal–Wallis test: H (2) = 12.309, *FDR-adjusted p* < 0.05).

Satisfaction with support for professional development and continuing education was also associated with several favorable responses. Mean scores for canceling leisure activities when faced with an unusually high workload were lowest among respondents who were satisfied or very satisfied with such support (mean: 3.56 ± 1.09), followed by neutral respondents (mean: 3.76 ± 1.28) and those who were dissatisfied or very dissatisfied (mean: 4.19 ± 1.13) (Kruskal–Wallis test: H (2) = 11.829, *FDR-adjusted p* < 0.05). The mean score for the ability to discuss difficult cases with more experienced colleagues was highest among satisfied or very satisfied respondents (mean: 4.38 ± 0.73), followed by neutral respondents (mean: 3.85 ± 1.03) and those who were dissatisfied or very dissatisfied (mean: 3.67 ± 1.20) (Kruskal–Wallis test: H (2) = 13.699, *FDR-adjusted p* < 0.05). Similarly, participation in training courses to remain up to date and increase self-confidence was highest among satisfied or very satisfied respondents (mean: 4.47 ± 0.68), compared with neutral (mean: 3.94 ± 0.86) and dissatisfied or very dissatisfied respondents (mean: 3.95 ± 0.96) (Kruskal–Wallis test: H (2) = 13.722, *FDR-adjusted p* < 0.05). Mean scores for team-based process optimization were also highest among satisfied or very satisfied respondents (mean: 3.62 ± 1.06), followed by neutral respondents (mean: 3.33 ± 1.19) and those who were dissatisfied or very dissatisfied (mean: 2.33 ± 1.12) (Kruskal–Wallis test: H (2) = 29.436, *FDR-adjusted p* < 0.0001). Perceived empathy or understanding from colleagues following adverse events was likewise highest among satisfied or very satisfied respondents (mean: 4.08 ± 0.89), followed by neutral respondents (mean: 3.85 ± 0.94) and those who were dissatisfied or very dissatisfied (mean: 3.26 ± 1.06) (Kruskal–Wallis test: H (2) = 17.069, *FDR-adjusted p* < 0.01).

#### 3.3.2 Emotional responses to adverse events

When adverse events occurred, stress was the predominant emotional reaction: 64/152 respondents (42.1%) reported often feeling stressed, and 38/152 (25.0%) reported always feeling stressed. More than half of the respondents (86/152; 56.6%) also frequently experienced self-doubt, with 48/152 (31.6%) reporting this often and 38/152 (25.0%) always. Sadness was reported often or always by 54/152 respondents (35.5%), while guilt and anxiety were reported often or always by 60/152 (39.4%) and 53/152 (34.9%), respectively. Anger was less common, with 43/152 respondents (28.2%) reporting it often or always, while 101/152 (66.5%) reported experiencing anger rarely or sometimes (Figure 3). Veterinarians with poor or very poor work-life balance reported experiencing stress after adverse events more frequently (29/34; 85.3% often or always) than those with fair (44/67; 65.7%) or excellent or good work-life balance (29/51; 56.9%) (χ^2^ = 19.382, *FDR-adjusted p* < 0.05). In contrast, veterinarians who were satisfied or very satisfied with support for professional development and continuing education reported stress after adverse events less frequently (45/77; 58.4% often or always) than neutral respondents (28/33; 84.8%) or those who were dissatisfied or very dissatisfied respondents (29/42; 69.0%) (χ^2^ = 14.259, *FDR-adjusted p* < 0.05). Marital status was significantly associated with anxiety after adverse events, with unmarried veterinarians reporting anxiety often or always more frequently than married respondents (42/96; 43.8% vs. 11/56; 19.6%; χ^2^ = 12.055, *FDR-adjusted p* < 0.05).

The duration of preoccupation after stressful events or challenging situations also varied. Approximately half of the respondents reported that they remained preoccupied with such events for a few days (78/152; 51.3%). A further 23/152 respondents (15.1%) reported thinking about such events for 1 week, while 21/152 (13.8%) remained preoccupied for 2–3 weeks. Preoccupation lasting only for the remainder of the day was reported by 15/152 respondents (9.9%), whereas longer-lasting preoccupation for 1 month (7/152; 4.6%), several months (7/152; 4.6%), or more than 1 year (1/152; 0.7%) was less common.

#### 3.3.3 Responses to mistakes and confidence-related indicators

Respondents generally reported a learning-oriented response to mistakes or adverse events, with high agreement that they actively sought ways to improve their skills and knowledge to prevent similar situations from recurring (mean: 4.32 ± 0.77) (Figure 4). Confidence-related responses were more moderate: respondents reported remaining confident in their knowledge and abilities despite mistakes (mean: 3.14 ± 1.08), while agreement was somewhat lower with the statement that mistakes can occur without significantly affecting confidence (mean: 2.95 ± 1.08).

**Figure 4:**
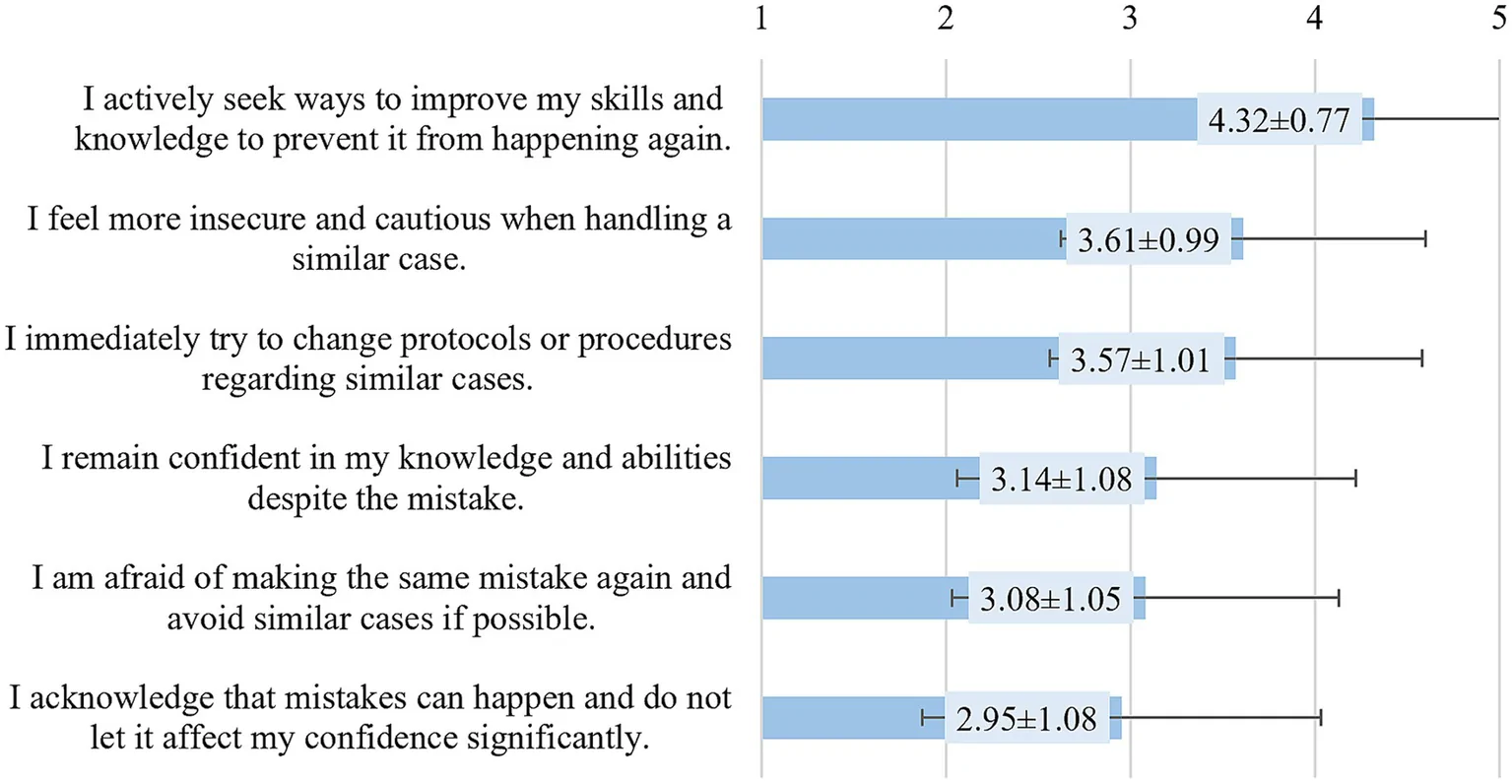
Female veterinarians’ emotional and professional responses to mistakes or adverse events (mean ± SD; *n* = 152). Responses were given on a five-point Likert scale, where 1 = strongly disagree, 2 = disagree, 3 = neither agree nor disagree, 4 = agree, and 5 = strongly agree.

Supplementary Tables 17–24 provide detailed results by socio-demographic and occupational variables. Professional position was associated with several responses to mistakes or adverse events. Employers reported changing protocols or procedures for similar cases more often (mean: 4.00 ± 0.72) than employed veterinarians (mean: 3.45 ± 1.05). Employers were also more likely to acknowledge that mistakes can happen without significantly affecting their confidence (mean: 3.38 ± 1.34) than employed veterinarians (mean: 2.83 ± 0.98). Similarly, employers reported greater confidence in their knowledge and abilities despite mistakes (mean: 3.75 ± 1.05) than employed veterinarians (mean: 2.98 ± 1.04) (Mann–Whitney U test: U = 1160.5, Z = −3.567, *FDR-adjusted p* < 0.01).

Professional experience was also associated with learning-oriented and confidence-related responses. Mean scores for active efforts to improve skills and knowledge were highest among veterinarians with 6–10 years of experience (mean: 4.59 ± 0.71), followed by those with 0–5 years (mean: 4.33 ± 0.75), more than 20 years (mean: 4.33 ± 0.72), and 11–20 years of experience (mean: 4.05 ± 0.73). Mean agreement that mistakes can occur without significantly affecting confidence was highest among veterinarians with more than 20 years of experience (mean: 3.80 ± 1.15), followed by those with 6–10 years (mean: 3.03 ± 1.03), 0–5 years (mean: 2.80 ± 0.98), and 11–20 years of experience (mean: 2.79 ± 1.15). Confidence in knowledge and abilities despite mistakes was also highest among veterinarians with more than 20 years of experience (mean: 3.87 ± 0.92), followed by those with 6–10 years (mean: 3.34 ± 1.13), 11–20 years (mean: 3.28 ± 1.08), and 0–5 years of experience (mean: 2.80 ± 1.00) (Kruskal–Wallis test: H (3) = 15.555, *FDR-adjusted p* < 0.05).

Work-life balance was associated with several emotional and confidence-related responses to mistakes or adverse events. Mean scores for fear of making the same mistake again and avoiding similar cases were highest among respondents with poor or very poor work-life balance (mean: 3.56 ± 0.99), followed by those with fair (mean: 2.97 ± 1.10) and excellent or good work-life balance (mean: 2.90 ± 0.94). Mean scores for feeling insecure and cautious when handling similar cases were also highest among respondents with poor or very poor work-life balance (mean: 3.97 ± 1.03), followed by respondents with excellent or good (mean: 3.63 ± 0.85) and fair work-life balance (mean: 3.42 ± 1.03). In contrast, agreement that mistakes can occur without significantly affecting confidence was highest among respondents with excellent or good work-life balance (mean: 3.10 ± 0.96), followed by those with fair (mean: 3.09 ± 1.04) and poor or very poor work-life balance (mean: 2.44 ± 1.21). Confidence in knowledge and abilities despite mistakes was also highest among respondents with excellent or good work-life balance (mean: 3.37 ± 1.02), followed by those with fair (mean: 3.18 ± 1.10) and poor or very poor work-life balance (mean: 2.74 ± 1.05).

### 3.4 Situation-specific coping strategies, career intentions, and support needs

#### 3.4.1 Coping responses to specific workplace challenges

Situation-specific coping strategies are summarized in Table 3. In relation to euthanasia-related emotional distress, respondents most frequently reported reminding themselves that euthanasia relieves the animal’s suffering (114/152; 75.0%) and reviewing the medical history or double-checking the equipment to feel confident in their decision (99/152; 65.1%). Collegial support was also commonly reported, with 63/152 respondents (41.4%) discussing such cases with colleagues for reassurance. In contrast, reflective writing or journaling (5/152; 3.3%) and professional counseling (2/152; 1.3%) were rarely reported. Overall, respondents primarily reported cognitive reframing and collegial reassurance, whereas structured reflective or therapeutic coping strategies were uncommon. Professional position was significantly associated with several euthanasia-related coping strategies. Employers reported seeking collegial reassurance less frequently than employed veterinarians (6/32; 18.8% vs. 57/120; 47.5%; χ^2^ = 8.604, *FDR-adjusted p* < 0.05), but reported using mindfulness or relaxation techniques (4/32; 12.5% vs. 4/120; 3.3%) and hobbies or leisure activities (10/32; 31.3% vs. 18/120; 15.0%) more frequently.

**Table 3:** Situation-specific coping strategies and emotional responses among female veterinarians (%; *n* = 152).

Clinical or work-related situation	Coping strategy or emotional response	Number of responses (*n*)	Percentage of responses (%)
Coping with euthanasia cases	I remind myself that the euthanasia will relieve the animal’s suffering	114	75.0%
	I review the medical history and double-check the equipment to feel confident in my decision	99	65.1%
	I talk to colleagues for reassurance that euthanasia is the appropriate decision	63	41.4%
	I do not specifically prepare myself	45	29.6%
	I engage in a hobby to unwind	28	18.4%
	I engage in physical exercise (e.g., running, swimming, gym workouts)	22	14.5%
	I use mindfulness or relaxation techniques, such as deep breathing or meditation, to calm myself	8	5.3%
	I engage in reflective writing or journaling	5	3.3%
	I try to distract myself by thinking about or engaging in non-work-related activities	3	2.0%
	I seek professional counseling	2	1.3%
	Other	10	6.6%
Client non-adherence	I feel frustrated, but I try to remain professional and focus on educating the client about the importance of the recommendation.	110	72.4%
	I accept that I cannot control the outcome and focus continuing to provide the best possible care.	74	48.7%
	I experience disappointment, but I remind myself that their decisions are ultimately out of my control and try maintain emotional distance to avoid burnout.	63	41.4%
	I feel empathy for the client’s situation and try to understand their perspective.	45	29.6%
	I feel motivated to reflect on my communication strategies and consider ways to improve my approach and encourage better adherence.	34	22.4%
	I feel frustrated and have difficulty managing my emotions.	11	7.2%
Unexpectedly high workload or emergency situations	Prioritizing tasks	137	90.1%
	Delegating tasks to colleagues	84	55.3%
	Seeking help from superiors	44	28.9%
	Taking short breaks	24	15.8%
	Practicing deep breathing or mindfulness	10	6.6%
	Other	9	5.9%
Negative feedback or complaints	Discussing the situation with colleagues	122	80.3%
	Reflecting on the feedback and making improvements	100	65.8%
	Letting it go and moving on	47	30.9%
	Seeking advice from a mentor	23	15.1%
	Using relaxation techniques	3	2.0%
	Other	8	5.3%
Aggressive or difficult animals	Ensuring that appropriate physical safety measures are in place	132	86.8%
	Using calming techniques to manage the animal	101	66.4%
	Seeking support from colleagues	70	46.1%
	Taking a moment to relax before proceeding	69	45.4%
	Using mental rehearsal or visualization	6	3.9%
	Other	4	2.6%

Multiple responses were permitted; therefore, percentages may total more than 100%.

Responses in the “Other” category for coping with euthanasia cases included talking to family or friends; lighting a memorial candle, making eye contact with colleagues, and taking a deep breath afterwards; and suppressing the experience until later in the day or at night.

Responses in the “Other” category for unexpectedly high workload or emergency situations included eating chocolate; talking to employees, family members, or colleagues; completing tasks one by one and working longer hours; complaining or venting with colleagues; smoking; feeling overwhelmed or frantic; and taking a moment to reflect and provide oneself with internal encouragement.

Responses in the “Other” category for negative feedback or complaints included speaking directly with clients; feeling annoyed; taking a clear position regarding unjustified feedback or unpaid invoices; talking to family or friends; persistent rumination and self-doubt; and keeping the experience to oneself.

Responses in the “Other” category for aggressive or difficult animals included consulting veterinary assistants about the most appropriate approach; indicating that the situation was not applicable to the respondent’s workplace; dealing only with animals that were already deceased; and administering sedatives before the veterinary visit.

Regarding client non-adherence to professional recommendations, respondents most frequently reported feeling frustrated but trying to remain professional and focusing on educating the client about the importance of the recommendation (110/152; 72.4%). Nearly half of the respondents reported accepting that they could not control the outcome and focusing on providing the best possible care (74/152; 48.7%). Age group was significantly associated with reported empathy toward clients in such situations. Veterinarians aged 23–34 years reported empathy for the client’s situation more frequently (28/66; 42.4%) than those aged 35–44 years (11/56; 19.6%) or older than 45 years (6/30; 20.0%) (χ^2^ = 9.199, *FDR-adjusted p* < 0.05).

In situations involving an unexpectedly high workload or an emergency, respondents mainly reported task-oriented coping strategies. Prioritizing tasks was the most frequently selected strategy (137/152; 90.1%), followed by delegating tasks to colleagues (84/152; 55.3%). Seeking help from superiors was reported by 44/152 respondents (28.9%) and differed significantly by age group and professional position. Veterinarians aged 23–34 years reported seeking help from superiors more frequently (27/66; 40.9%) than those aged 35–44 years (10/56; 17.9%) or older than 45 years (7/30; 23.3%) (χ^2^ = 8.400, *FDR-adjusted p* < 0.05). Similarly, employed veterinarians reported seeking help from superiors more frequently than employers (42/120; 35.0% vs. 2/32; 6.3%) (χ^2^ = 10.153, *FDR-adjusted p* < 0.01).

Work-life balance was also associated with coping strategies in situations involving an unexpectedly high workload or an emergency. Respondents with excellent or good work-life balance reported delegating tasks more frequently (33/51; 64.7%) than those with fair (39/67; 58.2%) or poor or very poor work-life balance (12/34; 35.3%) (χ^2^ = 7.558, *FDR-adjusted p* < 0.05). Taking short breaks was also reported more frequently by respondents with excellent or good work-life balance (15/51; 29.4%) than by those with fair (7/67; 10.4%) or poor or very poor work-life balance (2/34; 5.9%) (χ^2^ = 11.065, *FDR-adjusted p* < 0.05). Similarly, practicing deep breathing or mindfulness was more common among respondents with excellent or good work-life balance (7/51; 13.7%) than among those with fair (3/67; 4.5%) or poor or very poor work-life balance (0/34; 0.0%) (χ^2^ = 7.114, *FDR-adjusted p* < 0.05).

In response to negative feedback or complaints from clients, the most frequently reported coping strategy was discussing the situation with colleagues (122/152; 80.3%). Seeking advice from a mentor was reported by 23/152 respondents (15.1%) overall, but this strategy was more common among veterinarians aged 23–34 years (17/66; 25.8%) than among those aged 35–44 years (5/56; 8.9%) or older than 45 years (1/30; 3.3%) (χ^2^ = 10.733, *FDR-adjusted p* < 0.05). It was also more frequently reported by veterinarians with 0–5 years of professional experience (18/66; 27.3%) than by those with 6–10 years (2/32; 6.3%), 11–20 years (3/39; 7.7%), or more than 20 years of professional experience (0/15; 0.0%) (χ^2^ = 13.897, *FDR-adjusted p* < 0.05). Similarly, employed veterinarians reported discussing negative feedback or complaints with colleagues more frequently than employers (104/120; 86.7% vs. 18/32; 56.3%) (χ^2^ = 14.754, *FDR-adjusted p* < 0.01). When dealing with aggressive or difficult animals, respondents most frequently reported implementing physical safety measures (132/152; 86.8%) and using calming techniques to manage the animal (101/152; 66.4%).

#### 3.4.2 Career intentions and perceived support needs

Career intentions indicated that a substantial proportion of respondents had considered changing their professional path. Overall, 59/152 respondents (38.8%) reported that they had not considered leaving the veterinary field, whereas 40/152 (26.3%) had considered moving to another area of veterinary medicine and 53/152 (34.9%) had considered leaving the profession entirely. Professional position was significantly associated with career intentions. Employed veterinarians more frequently reported having considered either moving to another area of veterinary medicine or leaving the profession entirely than employers (81/120; 67.5% vs. 13/32; 40.6%) (χ^2^ = 7.306, *FDR-adjusted p* < 0.05). More specifically, consideration of leaving the veterinary profession entirely was more frequent among employed veterinarians than among employers (45/120; 37.5% vs. 8/32; 25.0%).

Supportive resources already implemented or desired by respondents, along with preferred training topics are summarized in Table 4. The most frequently selected supportive resources were improved work-life balance (97/152; 63.8%), additional staff or support (94/152; 61.8%), and improved workplace communication and feedback (89/152; 58.6%). Age group was significantly associated with the preference for improved workplace communication and feedback, which was more frequently selected by veterinarians aged 23–34 years (47/66; 71.2%) than by those aged 35–44 years (26/56; 46.4%) or older than 45 years (16/30; 53.3%) (χ^2^ = 8.087, *FDR-adjusted p* < 0.05). Marital status was associated with the preference for improved work-life balance, which was selected more often by unmarried respondents than by married respondents (67/96; 69.8% vs. 29/56; 51.8%).

**Table 4:** Implemented or desired resources and preferred training topics for stress management among female veterinarians (%; *n* = 152).

Support-related topic	Implemented or desired resource/preferred training	Number of responses (*n*)	Percentage of responses (%)
Implemented or desired changes/resources	Additional staff support	94	61.8%
	Improved work-life balance (e.g., setting boundaries, scheduling leisure time)	97	63.8%
	Improved workplace communication and feedback	89	58.6%
	Flexible work hours or reduced on-call shifts	78	51.3%
	Engaging in hobbies or recreational activities	73	48.0%
	Training in stress management and resilience	58	38.2%
	Changes to diet or sleep habits	46	30.3%
	Use of time management techniques	43	28.3%
	Participation in stress-management workshops or training	34	22.4%
	Regular professional counseling services	31	20.4%
	Establishing a regular exercise routine	29	19.1%
	Establishing a support network or seeking mentorship	25	16.4%
	Beginning mindfulness or meditation practice	23	15.1%
	I would not change anything	2	1.3%
	Other	5	3.3%
Preferred training or workshops	Communication skills for interactions with clients and colleagues	48	31.6%
	Emotional intelligence and resilience	41	27.0%
	Time management and prioritization	29	19.1%
	Stress management techniques	26	17.1%
	Mindfulness and meditation	6	3.9%
	Other	2	1.3%

Multiple responses were permitted; therefore, percentages may total more than 100%.

Responses in the “Other” category for implemented or desired changes/resources included receiving time off in lieu of overtime, changing jobs, greater recognition from supervisors, and salary increases. Responses in the “Other” category for preferred training or workshops included managing difficult clients and opportunities for exchange with colleagues.

Working hours and on-call duties were also associated with selected support needs and self-care practices. Weekly working hours were significantly associated with having begun practicing mindfulness or meditation, which was reported more frequently by veterinarians working 40 h or less per week than by those working more than 40 h per week (20/95; 21.1% vs. 3/57; 5.3%) (χ^2^ = 6.916, *FDR-adjusted p* < 0.05). The frequency of on-call shifts was significantly associated with the preference for regular professional counseling services as a supportive resource. This option was most frequently selected by veterinarians with one on-call shift per week (7/19; 36.8%), followed by those with on-call duties once per month (9/34; 26.5%) and two to three times per month (5/20; 25.0%). In contrast, it was selected less frequently by respondents without on-call duties (9/48; 18.8%) and by those with more than one on-call shift per week (1/31; 3.2%) (χ^2^ = 9.909, *FDR-adjusted p* < 0.05).

Regarding preferred training or workshop topics, respondents most frequently selected communication skills for interactions with clients and colleagues (48/152; 31.6%) and training in emotional intelligence and resilience (41/152; 27.0%).

## 4 Discussion

This study examined coping strategies used by female veterinarians in Germany when dealing with work-related stress and emotionally demanding workplace situations. Overall, workload and work-life balance showed the most consistent associations with occupational strain, while support for professional development, collegial relationships, and a supportive learning environment were associated with more favorable team-related and emotion-regulation indicators. These findings are consistent with previous research showing that workload, long working hours, work-life balance, and organizational support are important factors related to occupational stress, burnout, mental health, and job satisfaction in veterinary professionals (7, 9, 15, 17, 23, 28). At the same time, the present study adds a specific perspective on female veterinarians working in Germany by examining how these factors are reflected in coping strategies, recovery-related behaviors, and perceived support needs.

### 4.1 Stress management, recovery, and work-life balance

The findings of the present study suggest that high job demands combined with limited resources are associated with greater perceived stress, lower workload manageability, and less favorable coping patterns. Core occupational stressors, including long working hours and night or weekend shifts, have repeatedly been identified as important contributors to exhaustion and burnout in veterinary medicine (4–7, 9, 10). This is consistent with the present findings, in which extended working hours were associated with lower perceived workload manageability, more frequent stress or burnout symptoms, and fewer opportunities for conscious breaks and recovery.

Working more than 40 h per week was also associated with less frequent conscious breaks and lower engagement in leisure-related coping strategies (9, 28, 38, 42). This may indicate that extended working hours not only increase exposure to occupational demands, but also reduce access to recovery opportunities that are necessary for maintaining emotional well-being. Similarly, on-call responsibilities appeared to be linked to lower perceived capacity for emotion regulation, particularly when recovery time and schedule predictability were limited. These findings support the view that workload-related strain should not be understood solely as a matter of individual resilience, but also as a consequence of work organization and the availability of structural resources, with potential implications for both mental and physical health (7, 17).

Work-life balance emerged as one of the most consistent factors associated with stress management and coping. This aligns with previous findings indicating that work-life balance is an important contributor to job satisfaction, particularly among young and female veterinarians (28). In the present study, poorer work-life balance was associated with higher levels of stress, feeling overwhelmed, and carrying work-related stress home, while better work-life balance was associated with more frequent use of adaptive coping strategies, including social support, leisure activities, task delegation, short breaks, and mindfulness or breathing techniques. These findings suggest that work-life balance may facilitate recovery and promote the use of constructive coping strategies (9, 17, 38). However, the present data do not allow us to determine whether the observed differences reflect career-stage effects, such as the additional learning and adjustment demands faced by less experienced veterinarians, generational differences in expectations regarding work-life balance, or other workplace-related factors. Therefore, these findings should be interpreted cautiously and warrant further qualitative and longitudinal research.

The results also indicate that work-life balance is linked to the social and communicative dimensions of veterinary work. Veterinarians with better work-life balance reported more favorable perceptions of collegial support, client empathy, and team-based process optimization. In contrast, poorer work-life balance was associated with reduced participation in leisure activities and greater reliance on avoiding or ignoring stress. This pattern suggests that insufficient recovery may not only intensify perceived strain, but also reduce access to the emotional and social resources needed to manage stressful workplace interactions effectively (9, 17, 28, 38, 42).

The coping patterns observed in the present study are broadly consistent with previous literature. Veterinarians in this sample most frequently relied on informal social support and practical everyday coping strategies, while more formal, reflective, or therapeutic approaches, such as mindfulness practices and professional counseling, were used less often. Previous research indicates that women more often mobilize private contacts as a resource for stress management than men (20). This pattern was also reflected in the present women-only sample, in which talking to friends or family was the most frequently reported general stress-management strategy. At the same time, although earlier work suggests a greater willingness among women to seek professional counseling (23), the present findings provide a more nuanced picture, as only a minority of respondents reported using professional counseling or therapy. Because no male comparison group was included, gender differences cannot be assessed in this study. Nevertheless, the results indicate a strong reliance on informal and private support networks alongside relatively low uptake of formal psychological support. Potential explanations may include perceived stigma, barriers to access, or the limited integration of such services into routine veterinary practice; however, these factors were not directly assessed in the present study (7, 9).

Maladaptive coping has been described as especially common under chronic stress (8, 14, 15). Research focusing on female veterinarians has also reported frequent rumination, resignation, and self-blame, while positive coping mechanisms were used less often (8). The present findings partly mirror and extend this pattern. Avoidance and the tendency to ignore stress were evident in the sample and were more common among respondents with poorer work-life balance. At the same time, adaptive coping strategies, including social support, leisure activities, task prioritization, delegation, and collegial discussion, were also frequently reported. This suggests that maladaptive and adaptive coping strategies may coexist, particularly when high job demands are not sufficiently balanced by recovery opportunities and organizational support (8, 14, 15, 17, 18).

### 4.2 Workplace support, professional learning, and responses to adverse events

Organizational resources also appeared to play an important role. Satisfaction with support for professional development and continuing education, the ability to discuss difficult cases with colleagues, perceived empathy from colleagues, and team-based process optimization were all associated with more favorable indicators of stress regulation and professional confidence. Overall, these findings suggest that workplaces that actively foster professional learning, feedback, and collegial support may create environments in which veterinarians feel more competent, valued, and emotionally supported (17, 18, 39). This interpretation is consistent with existing evidence showing that clear communication, a positive team climate, collegial recognition, mentoring, and supportive team cultures are associated with higher job satisfaction, lower perceived stress, improved mental well-being, and more sustainable veterinary careers (2, 17, 18, 46).

Studies on resilience emphasize that self-efficacy, coping skills, and social resources are key protective factors (2, 15, 18, 19). This is consistent with the present findings regarding support for continuing education, a supportive feedback culture, collegial case discussions, and perceived team efficacy. These resources may strengthen veterinarians’ confidence, facilitate emotional processing, and support more adaptive coping responses when facing stressful or emotionally demanding workplace situations (17–19, 26, 39).

In the present study, higher scores for perceived team efficiency and process optimization among more experienced veterinarians may reflect both greater influence within the team and more realistic expectations concerning teamwork and workflow improvement. Similarly, employers reported greater confidence when dealing with professional errors, which may be related to broader professional experience, higher autonomy, and coping mechanisms acquired through professional and managerial responsibility. Age-related patterns were descriptively similar to those observed for professional experience, which is consistent with previous findings indicating better stress management and self-care skills among older veterinarians and those in more autonomous or leadership-related positions (19, 23). However, age, professional experience, and employer status are closely related characteristics, and their independent contributions cannot be determined from the present analyses.

### 4.3 Career stage, support-seeking, and professional retention

The findings on career-stage-specific differences are largely consistent with previous research. Early-career, younger and less experienced veterinarians more often reported feeling overwhelmed, and younger respondents more frequently relied on hierarchical or mentoring support. This pattern may reflect the “reality shock” described among newly graduated veterinarians, which has been linked to adverse mental health outcomes, particularly among young veterinarians and young women (23, 34). In contrast, greater professional experience and employer status were associated with greater self-confidence, more favorable team- and process-related indicators, and lower levels of feeling overwhelmed, which may reflect the gradual development of stress-management and self-care skills, greater autonomy, or both (2, 19, 23).

The observed differences in support-seeking behavior may reflect both career-stage and experiential factors. Younger and less experienced veterinarians more frequently relied on mentoring, support from superiors, and collegial reassurance, suggesting that supervisory guidance may play an important role in stress management during the early stages of a veterinary career (17–19, 34). With increasing professional experience, reliance on formal mentoring or hierarchical support appeared to decrease, which may indicate that coping mechanisms become more autonomous over time. However, this pattern should be interpreted cautiously, as greater independence may also be accompanied by a less willingness among more experienced veterinarians or employers to seek help when needed. Therefore, supportive and psychologically safe workplace cultures should be maintained across all career stages, not only for early-career veterinarians (17, 23).

Against this background, early-career support structures may be particularly useful for helping young veterinarians reach greater confidence and professional stability more quickly, while reducing the risk of prolonged periods of psychological strain or considering leaving the profession. Since veterinary work is still widely perceived as meaningful and rewarding (17, 26, 47), supporting adaptive coping and professional well-being across all career stages is important for promoting long-term commitment to the profession and overall well-being (19). These findings are also relevant in light of recent research on veterinary career intentions among Hungarian students, which suggests that motivational characteristics may already shape future professional commitment before entering the labor market (48).

### 4.4 Practical implications and future research

The findings of the present study should be interpreted in light of existing recommendations suggesting that veterinary mental health should be addressed through individual, social, and organizational interventions. At the individual level, training in emotional awareness, emotion regulation, adaptive coping, mindfulness, physical activity, and self-care routines may help strengthen personal resources (14, 16, 17, 19, 29, 32). At the social level, peer mentoring, collegial debriefings, structured discussions after difficult cases, ethics reflection groups, and efforts to reduce stigma surrounding psychological support may facilitate timely emotional processing and reduce isolation (1, 3, 16, 18, 19, 26, 29, 39). At the organizational level, predictable working hours, protected breaks, adequate leave, fair pay, supervision, coaching, flexible working models, mental health programs, and psychologically safe workplace cultures may support long-term mental well-being and job retention (7, 9, 14, 15, 17, 19, 23, 26, 28, 37). However, individual coping strategies may have limited effectiveness if they are not supported by appropriate organizational conditions. This is particularly relevant to the present findings, as workload, work-life balance, and satisfaction with support for professional development and continuing education were associated with several indicators of stress management and coping (9, 17, 28, 38, 42).

The findings suggest that quantitative work demands should be managed in a structured manner and that recovery periods should be actively protected (4, 8, 27). In practice, this means that working hours should be kept within manageable limits and that unavoidable overtime should be compensated promptly rather than allowed to accumulate. Although the present findings showed less favorable outcomes among veterinarians working more than 40 h per week, the cross-sectional design does not establish a specific causal threshold for working time. Breaks should be integrated into work schedules and supported by adequate cover arrangements, so that acute cases do not continuously displace planned rest periods. Adequate staffing is also essential to prevent excessive on-call duties and to distribute workload more evenly across team members. Transparent recording of working hours, breaks, overtime, and on-call duties may further help identify recurring planning problems and improve work organization (17, 28, 38, 42, 49–51).

The findings also suggest that work-life balance and working-time structures are closely associated with the ability to use adaptive coping strategies. Unmarried veterinarians more frequently selected improved work-life balance as a desired resource, which may reflect differences in perceived support needs or available personal resources. Similarly, veterinarians working 40 h or less per week more often reported having begun practicing mindfulness or meditation. This may indicate that shorter or more manageable working hours are associated with greater opportunity for time-dependent self-care strategies. However, the present study did not distinguish between full-time and part-time employment within this group, nor did it assess the reasons for working 40 h or less per week. Therefore, it cannot be determined whether this pattern reflects better organizational planning, a previous stress-related reduction in working hours, family-related circumstances, or other individual or workplace factors. These results underline that self-care should not be treated solely as an individual responsibility, but should be supported by realistic schedules and workplace cultures that make recovery possible (17, 38, 42, 52).

The benefits of a workplace culture that promotes professional learning and emotional processing were also suggested by the present findings. Regular structured case discussions may help teams reflect not only on medical aspects, but also on communication, ethical challenges, and the emotional consequences of difficult cases. Such discussions may contribute to professional confidence, shared learning, and stronger team cohesion. Additional peer mentoring and supervision can provide reliable points of contact, especially for younger and less experienced colleagues (8, 14, 15, 17). Fixed mentoring pairs, regular meetings, and additional support after stressful events may help build trust and facilitate timely professional and emotional support (17, 18).

The low uptake of professional counseling and mindfulness-based strategies in the present sample suggests that structured psychological or self-reflective coping approaches may still be less accessible, less normalized, or less prioritized than informal support and practical coping strategies (17, 23, 53, 54). Although many veterinarians reported discussing stress and emotional difficulties with friends, family members, or colleagues, professional support may provide an additional resource for recognizing maladaptive coping patterns and developing healthier long-term strategies. However, the present study did not assess the reasons for the limited use of such services. Improving access to professional counseling and supervision and reducing stigma surrounding their use may therefore support a more sustainable coping culture within veterinary workplaces (17, 53, 54).

The association between on-call frequency and interest in regular professional counseling services further supports the potential value of accessible mental health resources. Interest was highest among veterinarians with one on-call shift per week and lower among both those without on-call duties and those with more than one on-call shift per week. This non-linear pattern may reflect differences in perceived need, available time, access, or the normalization of strain under highly demanding schedules. Because these explanations were not directly assessed, they remain tentative. These findings nevertheless support making counseling and supervision readily accessible to veterinarians exposed to recurrent emotionally demanding or unpredictable work schedules (9, 17, 53, 54).

Recovery may also be supported through brief practical routines, such as short pauses, breathing exercises, walking, or hydration breaks, provided that sufficient time and a psychologically safe work environment are available (14, 17, 42). Such measures should complement, rather than substitute for, broader organizational efforts to manage workload and protect recovery time. Taken together, these findings suggest that effective communication, structured professional support, and workplace-level prevention strategies may help reduce occupational strain and support sustainable veterinary careers (9, 17, 38, 51, 53–55).

Future research should build on these exploratory findings using larger and more representative samples, including male and gender-diverse veterinarians. Longitudinal, qualitative, and intervention studies would be useful to clarify how workload, work-life balance, workplace support, and coping strategies interact over time, and to evaluate which workplace-based support measures are most effective in practice.

### 4.5 Limitations

Several limitations should be considered when interpreting the findings of this study. First, the study used a cross-sectional design based on self-reported data. Therefore, the results describe associations between demographic, occupational, and coping-related variables, but no conclusions can be drawn regarding causality or temporal relationships. In addition, common method bias may have occurred because all variables were collected from the same respondents using the same questionnaire at the same point in time. Recalling bias, reporting bias, social desirability bias, and individual differences in response styles may also have influenced the responses.

Second, participants were recruited through non-probability convenience sampling via online channels, including social media, veterinary mailing lists, and veterinary newsletters. As a result, the sample cannot be considered representative of all female veterinarians in Germany. Response rates could not be calculated, and no weighting according to population characteristics was performed. Self-selection bias is also possible, as veterinarians with stronger experiences or opinions related to stress, coping, emotional burden, work-life balance, or professional support may have been more likely to participate. Consequently, the findings should be interpreted as exploratory associations rather than as representative estimates for the wider population of female veterinarians in Germany.

Third, the analytical sample was limited to female veterinarians. Although this enabled the study to focus specifically on the experiences of women in an increasingly female-dominated profession, it limits the transferability of the findings to male veterinarians. This is particularly relevant because older generations in veterinary medicine have historically been more male-dominated. As a result, the perspectives of older male veterinarians, particularly regarding autonomy, help-seeking behavior, stress management, and coping with professional responsibility, were not captured in the present study.

Fourth, although the questionnaire was developed on the basis of a literature review and pilot-tested for clarity, relevance, and comprehensibility, it was not formally psychometrically validated. The study did not use validated composite scales for burnout, stress, coping, mental health, or work-life balance, but relied on individual self-reported questionnaire items. Therefore, the results should be interpreted as self-reported perceptions and experiences related to stress and coping, rather than as clinically validated measures of psychological distress or burnout. The use of individual Likert-scale items may also have limited measurement precision and comparability with studies using validated instruments.

Finally, the analyses were exploratory and primarily univariable. Although associations between demographic or occupational factors and coping-related responses were examined, residual confounding by factors such as type of practice, workload intensity, income, workplace culture, availability of support, previous mental health history, personality traits, or personal circumstances cannot be excluded. Moreover, closely related variables such as age, professional experience, and employer status were not examined simultaneously in multivariable models, and their independent associations therefore could not be determined. In addition, the large number of statistical comparisons and the absence of adjustment for multiple testing increase the possibility of chance findings. Some subgroup analyses may also have been based on small cell counts, reducing statistical power and the stability of the estimates. Overall, the findings should therefore be interpreted as exploratory associations in a convenience sample of female veterinarians in Germany, rather than as causal, representative, or fully generalizable conclusions.

## 5 Conclusion

This study provides insight into occupational stress, work-life balance, and coping among female veterinarians in Germany. The findings suggest that workload, work-life balance, support for professional development and continuing education, and workplace culture are associated with coping patterns and perceived emotional strain. Respondents most frequently relied on social support, practical problem-solving, and cognitive reframing, whereas professional counseling and mindfulness-based strategies were less commonly used. Poorer work-life balance was associated with higher levels of perceived stress and more avoidance-based coping, while supportive professional environments were associated with more favorable coping patterns, confidence-related and team-related indicators. These findings underline the importance of workplace-based prevention strategies, including predictable working hours, protected breaks, mentoring, collegial case discussions, and accessible psychological support. Strengthening both individual coping resources and organizational support structures may help promote more sustainable veterinary careers and better overall well-being.

## Data Availability

The raw data supporting the conclusions of this article will be made available by the authors, without undue reservation.
