# Supplementary material for: Occupational stress, work-life balance, and coping among female veterinarians in Germany
Source: Front Vet Sci. 2026 Sep 18;13:1936424. doi: 10.3389/fvets.2026.1936424 (PMC13631423; doi:10.3389/fvets.2026.1936424)
Supplement: Supplementary file 1 [file Data_Sheet_1.PDF]

Supplementary Material

1 Supplementary Data

Our research focuses on investigating the strategies used by veterinarians in Germany to managing stress and emotional difficulties associated with their profession. It aims to identify the specific methods they rely on in terms of self-care, particularly when dealing with high levels of occupational stress. Additionally, the findings are intended to inform the development of practical interventions by mental health professionals and veterinary workplaces to support the well-being of veterinary practitioners.

The questionnaire is completely anonymous and will take a maximum of 15 minutes to complete. All responses will be kept confidential, and the data will be analyzed and reported only in aggregate form to protect participants' privacy. Participation is voluntary, and you may discontinue the questionnaire at any time before submitting your responses without any consequences.

Your input is invaluable to this research, and we greatly appreciate your time and honesty in responding to the questions.

Thank you for your participation!

SOCIODEMOGRAPHIC QUESTIONS

Q1: Which age group do you belong to?

- 23-34 years
- 35-44 years
- 45-54 years
- 55-64 years
- 65-74 years
- older than 74 years

Q2: What is your gender?

- female
- male
- prefer not to say

Q3: What is your current marital status?

- unmarried
- married
- divorced
- widow
- other _______________

Q4: In which federal state do you live and work?

- Baden-Württemberg
- Bayern
- Berlin
- Brandenburg
- Bremen
- Hamburg
- Hessen
- Mecklenburg-Vorpommern
- Niedersachsen
- Nordrhein-Westfalen
- Rheinland-Pfalz
- Saarland
- Sachsen-Anhalt
- Sachsen
- Schleswig-Holstein
- Thüringen

Q5: What is your place of residence according to the number of residents?

- city (> 100.000)
- middle town (20.001-100.000)
- small town (5.000-20.000)
- rural community (< 5.000)

Q6: In which field of veterinary medicine are you working?

- small animal veterinarian
- equine veterinarian
- farm animal veterinarian (cattle, pig, poultry)
- exotic animal veterinarian
- authorities/state vet
- pharmaceutical industry
- research/education
- laboratory diagnostics
- other: _________________________

Q7: What describes your average weekly work hours the best?

- less than 20 hours
- 20-30 hours
- 31-40 hours
- 41-50 hours
- 51-60 hours
- more than 60 hours

Q8: Are you employed or employer?

- employed

- employer

Q9: How many years of professional practice experience do you have?

- < 1 year
- 1-5 years
- 6-10 years
- 11-20 years
- > 20 years

Q10: Are you obligated to have on-call shifts?

- No
- Yes, cc. once a month
- Yes, 2-3x per month
- Yes, once a week
- Yes, more than once a week

Q 11: How would you describe your general health status?

- Excellent
- Good
- Fair
- Poor
- Very poor

Work Environment, Emotional Awareness and Stress Management Skills

Likert 1-5 scale: Please select on a scale from 1- 5 how much do you agree with the following statements where 1 = strongly disagree, 2 = disagree, 3 = neither agree nor disagree, 4 = agree, 5 = strongly agree.

- Q12: I am aware of my feelings and emotions as they are happening.
- Q13: I can effectively manage my emotions and set boundaries when faced with challenging cases or unfair treatment.
- Q14: I consciously take breaks and pauses during which I don't dwell on work and potential stressors.
- Q15: I frequently carry work-related stress home, as I find myself preoccupied with it even after working hours.
- Q16: I am able to identify stressors in my work environment.
- Q17: I sometimes feel overwhelmed by the demands and responsibilities of my job as a veterinarian.
- Q18: I have close personal relationship to a partner/friends/family/colleagues.
- Q19: It is easy for me to talk about my emotions.
- Q20: I am aware of my emotional comfort zone, i.e. I know how to experience and express emotions in a way they are comfortable for me.
- Q 21: I feel that my workload is manageable.

- Q 22: I often feel stress or experience burnout symptoms (e.g., emotional exhaustion, depersonalization, reduced personal accomplishment).

Q23: How do you rate your work-life balance?

- Excellent
- Good
- Fair
- Poor
- Very poor

Q24: How satisfied are you with the support provided for professional development and continuing education in your workplace?

- Very satisfied
- Satisfied
- Neutral
- Dissatisfied
- Very dissatisfied

Connection between Stress and Emotional Coping Strategies

Q25: Which of the following stress management techniques do you use regularly? (You may choose more than one answer!)

- Physical exercise (e.g., running, swimming, gym workouts)
- Meditation or mindfulness practices
- Talking to friends or family
- Professional counseling or therapy
- Hobbies or leisure activities
- Drinking alcohol
- Avoidance or ignoring the stress
- Other: ___________________________

Q26: How often do you take breaks during your workday?

- Every hour
- Every 2-3 hours
- Once or twice a day
- Rarely
- Never

The following statements are about how veterinarians deal with typical stressors in the veterinary field.
Likert 1-5 scale: Please select on a scale from 1- 5 how much do you agree with the following statements where 1 = strongly disagree, 2 = disagree, 3 = neither agree nor disagree, 4 = agree, 5 = strongly agree.

- Q27: I cancel leisure activities when faced with an unusually high workload.

- Q28: When a client rises his voice against me it affects me in a long term.
- Q29: I can discuss difficult cases with other (more experienced) colleagues.
- Q30: I take part in training courses to be up to date and feel more self-confident.
- Q31: My team works on optimizing processes to work more efficient.
- Q32: I perceive empathy or understanding from the client's side towards me in case adverse effects happen.
- Q33: I perceive empathy or understanding from colleagues towards me in case adverse effects happen.

Q34: Rate the following emotions in term of their occurrence for you in case any adverse effect happens, where 1 = Never, 2 = Rarely, 3 = Sometimes, 4 = Often, 5 = Always.

- Guilt
- Anxiety
- Self-doubt
- Anger
- Sadness
- Stress

Q35: How long does it typically take for a stressful event or challenging situation at work to no longer occupy your thoughts?

- The rest of the day
- A few days
- One week
- 2-3 weeks
- One month
- Several months
- More than a year

<u>Specific Situations and Emotional Responses</u>

Please indicate how much you agree with the following statements regarding your reaction to a stressful or challenging situation at work?
Likert 1-5 scale: Please select on a scale from 1- 5 how much do you agree with the following statements where 1 = strongly disagree, 2 = disagree, 3 = neither agree nor disagree, 4 = agree, 5 = strongly agree.

- Q36: I am afraid of making the same mistake again and avoid similar cases if possible.
- Q37: I feel more insecure and cautious when handling a similar case.
- Q38: I immediately try to change protocols or procedures regarding similar cases.
- Q39: I actively seek ways to improve my skills and knowledge to prevent it from happening again.
- Q40: I acknowledge that mistakes can happen and do not let it affect my confidence significantly.
- Q41: I remain confident in my knowledge and abilities despite the mistake.

Q42: How do you mentally prepare yourself and cope with emotional distress when dealing with euthanasia cases? (You may choose more than one answer!)

- Mindfulness and relaxation techniques like deep breathing/meditation to calm down
- I review the medical history and double-check the equipment to feel confident in my decision
- I talk to colleagues to get support that the euthanasia is the right decision
- I remind myself that the euthanasia will relieve the animal from suffering
- I try to distract myself by thinking about or performing non-work-related topics
- I engage in reflective writing or journaling
- I seek professional counseling
- I engage in a hobby to unwind
- I practice physical exercise (e.g., running, swimming, gym workouts)
- I do not really prepare myself
- Other: _________________________

Q43: How would you describe your emotional response when clients not adhere to your professional recommendations? (You may choose more than one answer!)

- I feel empathy for the client's situation and try to understand their perspective.
- I feel motivated to reflect on my communication strategies and consider ways to improve my approach to ensure better client compliance.
- I feel frustrated, but I try to remain professional and focus on educating the client about the importance of the advice.
- I accept that I cannot control the outcome and focus on maintaining my resilience and continuing to provide the best care possible.
- I experience disappointment, but I remind myself that their decisions are ultimately out of my control and try to detach myself to avoid emotional burnout.
- I feel frustrated and cannot cope with my emotions very well.

Q44: How do you manage stress when faced with an unexpected high workload or emergency situations? (You may choose more than one answer!)

- Prioritizing tasks
- Delegating tasks to colleagues
- Taking short breaks
- Seeking help from superiors
- Practicing deep breathing or mindfulness
- Other: _________________________

Q45: How do you cope with negative feedback or complaints from clients? (You may choose more than one answer!)

- Discussing it with colleagues
- Seeking advice from a mentor
- Reflecting on the feedback and making improvements
- Letting it go and moving on
- Using relaxation techniques
- Other: _________________________

Q46: What strategies do you use to prepare yourself mentally and emotionally for dealing with aggressive or difficult animals? (You may choose more than one answer!)

- Mental rehearsal or visualization
- Seeking support from colleagues
- Ensuring physical safety measures are in place
- Using calming techniques for the animal
- Taking a moment to relax before proceeding
- Other: ___________________________

Q47: Have you ever considered to leave the veterinary field?

- No
- Yes, to change to another field of veterinary medicine
- Yes, to leave the veterinary field completely

Reflective and Improvement-Oriented Questions and Potential Solutions

Q48: What changes or resources have you implemented, or would you like to implement to better manage stress and emotional well-being in your practice? (You may choose more than one answer!)

- More staff or support
- Training in stress management and resilience
- Regular professional counseling services
- Improved workplace communication and feedback
- Flexible work hours or reduced on-call shifts
- Started practicing mindfulness or meditation
- Implemented regular exercise routine
- Improved work-life balance (e.g., setting boundaries, scheduling leisure time)
- Established a support network or sought mentorship
- Attended stress management workshops or training
- Made changes in diet or sleep habits
- Engaged in hobbies or recreational activities
- Implemented time management techniques
- Other: ___________________________

Q49: What type of training or workshops would you find most beneficial for managing stress and emotions in your profession?

- Stress management techniques
- Mindfulness and meditation
- Communication skills with clients and colleagues
- Time management and prioritization
- Emotional intelligence and resilience
- Other: ___________________________

Q50: If you have any additional comments regarding anything mentioned in the questionnaire, please add them below: ____

Thank you for your valuable contribution!

2 Supplementary Tables

Supplementary Table 1. Female veterinarians' perceptions of their emotional awareness and stress management skills according to age group (n = 152).

Statements (n=11)	Age group: 23-34 years (n=66)	35-44 years (n=56)	>45 years (n=30)	Pearson's chi-square (χ2) test: χ^2 (8)	p-value	FDR-adjusted p	Cramer's V Value	Kruskal Wallis: H (2)	p-value	FDR-adjusted p
	Mean ± SD									
I am aware of my feelings and emotions as they are happening.	3.88 ± 0.73	4.18 ± 0.72	4.03 ± 0.93	9.347	0.155	0.446	0.248	5.239	0.073	0.291
I can effectively manage my emotions and set boundaries when faced with challenging cases or unfair treatment.	2.83 ± 0.97	3.04 ± 0.89	3.07 ± 1.02	6.671	0.573	0.777	0.209	2.340	0.310	0.587
I consciously take breaks and pauses during which I don't dwell on work and potential stressors.	2.79 ± 1.17	3.09 ± 1.30	3.07 ±1.14	5.991	0.648	0.835	0.199	2.018	0.365	0.622
I frequently carry work-related stress home, as I find myself preoccupied with it even after working hours.	3.98 ± 1.06	3.84 ± 1.04	4.17 ± 1.02	4.326	0.827	0.897	0.169	2.403	0.301	0.574
I am able to identify stressors in my work environment.	3.94 ± 0.74	3.88 ± 0.79	3.87 ± 0.73	5.077	0.534	0.747	0.183	0.565	0.754	0.877
I sometimes feel overwhelmed by the demands and responsibilities of my job as a veterinarian.	3.94 ± 1.04	3.46 ± 1.18	3.67 ± 0.88	10.979	0.203	0.495	0.269	6.540	***<0.05****	0.192
I have close personal relationship to a partner/friends/family/colleagues.	4.44 ± 0.84	4.43 ± 0.97	4.33 ± 0.84	4.449	0.814	0.892	0.171	1.163	0.559	0.761
It is easy for me to talk about my emotions.	3.48 ± 1.09	3.46 ± 1.03	3.63 ± 1.25	5.589	0.693	0.856	0.192	0.884	0.643	0.835
I am aware of my emotional comfort zone, i.e. I know how to experience and express emotions in a way they are comfortable for me.	3.06 ± 0.89	3.34 ± 1.00	3.50 ± 1.01	10.629	0.224	0.502	0.264	5.149	0.076	0.299
I feel that my workload is manageable.	2.82 ± 1.08	2.93 ± 1.02	3.20 ± 1.19	5.675	0.684	0.852	0.193	2.677	0.262	0.534
I often feel stress or experience burnout symptoms (e.g., emotional exhaustion, depersonalization, reduced personal accomplishment).	3.45 ± 1.15	2.88 ± 1.25	3.23 ± 1.19	8.890	0.352	0.613	0.242	6.491	***<0.05****	0.194

An asterisk (*) denotes a statistically significant association ($p \leq 0.05$). Statistical significance was interpreted based on the FDR-adjusted p-values.

Supplementary Table 2. Female veterinarians' perceptions of their emotional awareness and stress management skills according to marital status (n = 152).

Statements (n=11)	Marital status									
	Unmarried (n=96)	Married (n=56)	Pearson's chi-square (χ2) test			Cramer's V Value	Mann-Whitney			
	Mean ± SD		χ² (4)	p-value	FDR-adjusted p		U	Z	p-value	FDR-adjusted p
I am aware of my feelings and emotions as they are happening.	4.04 ± 0.78	3.98 ± 0.77	0.643	0.887	0.930	0.065	2578.5	-0.462	0.644	0.835
I can effectively manage my emotions and set boundaries when faced with challenging cases or unfair treatment.	2.92 ± 0.98	3.02 ± 0.90	5.107	0.276	0.556	0.183	2555.5	-0.530	0.596	0.802
I consciously take breaks and pauses during which I don't dwell on work and potential stressors.	2.93 ± 1.28	3.00 ± 1.11	4.642	0.326	0.596	0.175	2574.0	-0.447	0.655	0.843
I frequently carry work-related stress home, as I find myself preoccupied with it even after working hours.	3.92 ± 1.07	4.05 ± 1.00	2.512	0.642	0.835	0.129	2511.5	-1.765	0.478	0.716
I am able to identify stressors in my work environment.	3.98 ± 0.74	3.77 ± 0.73	3.841	0.279	0.557	0.159	2266.5	-1.765	0.078	0.302
I sometimes feel overwhelmed by the demands and responsibilities of my job as a veterinarian.	3.76 ± 1.06	3.63 ± 1.11	4.273	0.370	0.625	0.168	2473.0	-0.862	***<0.05****	0.634
I have close personal relationship to a partner/friends/family/colleagues.	4.33 ± 0.98	4.55 ± 0.71	2.653	0.617	0.815	0.132	2413.0	-1.203	0.229	0.506
It is easy for me to talk about my emotions.	3.41 ± 1.10	3.68 ± 1.06	4.104	0.392	0.635	0.164	2308.5	-1.502	0.133	0.413
I am aware of my emotional comfort zone, i.e. I know how to experience and express emotions in a way they are comfortable for me.	3.23 ± 1.01	3.29 ± 0.89	2.094	0.718	0.862	0.117	2634.0	-0.216	0.829	0.898
I feel that my workload is manageable.	2.85 ± 1.11	3.07 ± 1.04	2.112	0.715	0.861	0.118	2373.5	-1.246	0.213	0.495
I often feel stress or experience burnout symptoms (e.g., emotional exhaustion, depersonalization, reduced personal accomplishment).	3.26 ± 1.23	3.09 ± 1.20	3.127	0.537	0.747	0.143	2445.5	-0.953	0.341	0.610

Supplementary Table 3. Female veterinarians' perceptions of their emotional awareness and stress management skills according to weekly working hours (n = 152).

Statements (n=11)	Weekly working hours									
	≤ 40 h (n=95)	> 40 h (n=57)	Pearson's chi-square (χ2) test			Cramer's V Value	Mann- Whitney			
	Mean ± SD		χ^2 (4)	p value	FDR-adjusted p		U	Z	p-value	FDR-adjusted p
I am aware of my feelings and emotions as they are happening.	3.99 ± 0.83	4.07 ± 0.68	2.338	0.505	0.738	0.124	2626.0	-0.441	0.732	0.867
I can effectively manage my emotions and set boundaries when faced with challenging cases or unfair treatment.	2.94 ± 0.99	2.98 ± 0.90	2.056	0.726	0.867	0.116	2628.5	-0.315	0.753	0.877
I consciously take breaks and pauses during which I don't dwell on work and potential stressors.	3.15 ± 1.19	2.63 ± 1.19	9.738	***<0.05****	0.211	0.253	2056.5	-2.545	***<0.05****	0.097
I frequently carry work-related stress home, as I find myself preoccupied with it even after working hours.	3.85 ± 1.11	4.16 ± 0.90	4.754	0.314	0.587	0.177	2332.0	-1.505	0.132	0.413
I am able to identify stressors in my work environment.	3.84 ± 0.78	4.00 ± 0.71	1.863	0.600	0.803	0.111	2417.5	-1.210	0.226	0.506
I sometimes feel overwhelmed by the demands and responsibilities of my job as a veterinarian.	3.66 ± 1.04	3.79 ± 1.15	9.904	***<0.05****	0.207	0.255	2427.5	-1.119	0.263	0.534
I have close personal relationship to a partner/friends/family/colleagues.	4.39 ± 0.96	4.46 ± 0.76	3.012	0.556	0.760	0.141	2687.5	-0.087	0.931	0.962
It is easy for me to talk about my emotions.	3.52 ± 1.14	3.49 ± 1.02	4.483	0.345	0.610	0.172	2610.5	-0.382	0.702	0.861
I am aware of my emotional comfort zone, i.e. I know how to experience and express emotions in a way they are comfortable for me.	3.26 ± 0.96	3.23 ± 0.98	0.489	0.975	0.987	0.057	2638.5	-0.276	0.783	0.892
I feel that my workload is manageable.	3.19 ± 1.07	2.51 ± 0.98	18.094	***<0.001****	***<0.05****	0.345	1717.0	-3.909	***<0.0001****	***<0.001****
I often feel stress or experience burnout symptoms (e.g., emotional exhaustion, depersonalization, reduced personal accomplishment).	3.04 ± 1.18	3.46 ± 1.24	5.877	0.209	0.495	0.197	2174.5	-2.086	***<0.05****	0.192

An asterisk (*) denotes a statistically significant association (p ≤ 0.05). Statistical significance was interpreted based on the FDR-adjusted p-values.

Supplementary Table 4. Female veterinarians' perceptions of their emotional awareness and stress management skills according to professional position (n = 152).

**Statements (n=11)**	**Position**									
	**Employed (n=120)**	**Employer (n=32)**	**Pearson's chi-square (χ2) test**			**Cramer's V Value**	**Mann- Whitney**			
	**Mean ± SD**		**χ² (4)**	**p-value**	**FDR-adjusted p**		**U**	**Z**	**p-value**	**FDR-adjusted p**
I am aware of my feelings and emotions as they are happening.	3.98 ± 0.80	4.19 ± 0.64	2.467	0.481	0.716	0.127	1680.0	-1.197	0.231	0.506
I can effectively manage my emotions and set boundaries when faced with challenging cases or unfair treatment.	2.88 ± 0.94	3.25 ± 0.95	6.276	0.179	0.475	0.203	1464.0	-2.159	***<0.05****	0.170
I consciously take breaks and pauses during which I don't dwell on work and potential stressors.	2.84 ± 1.20	3.38 ± 1.19	4.999	0.287	0.560	0.181	1444.0	-2.21	***<0.05****	0.161
I frequently carry work-related stress home, as I find myself preoccupied with it even after working hours.	3.91 ± 1.08	4.19 ± 0.90	2.324	0.676	0.852	0.124	1669.0	-1.194	0.232	0.506
I am able to identify stressors in my work environment.	3.93 ± 0.74	3.78 ± 0.79	10.834	***<0.05****	0.100	0.267	1656.5	-1.305	0.192	0.490
I sometimes feel overwhelmed by the demands and responsibilities of my job as a veterinarian.	3.75 ± 1.08	3.56 ± 1.08	3.254	0.516	0.742	0.146	1702.0	-1.034	0.301	0.574
I have close personal relationship to a partner/friends/family/colleagues.	4.38 ± 0.90	4.56 ± 0.84	3.651	0.455	0.702	0.155	1635.5	-1.472	0.141	0.418
It is easy for me to talk about my emotions.	3.45 ± 1.09	3.72 ± 1.09	4.480	0.345	0.610	0.172	1677.0	-1.138	0.255	0.532
I am aware of my emotional comfort zone, i.e. I know how to experience and express emotions in a way they are comfortable for me.	3.15 ± 0.94	3.63 ± 0.98	12.443	***<0.05****	0.104	0.286	1451.5	-2.222	***<0.05****	0.159
I feel that my workload is manageable.	2.90 ± 1.08	3.06 ± 1.11	0.594	0.964	0.986	0.063	1763.0	-0.736	0.462	0.709
I often feel stress or experience burnout symptoms (e.g., emotional exhaustion, depersonalization, reduced personal accomplishment).	3.27 ± 1.24	2.94 ± 1.13	6.996	0.136	0.413	0.215	1736.0	-1.315	0.188	0.490

An asterisk (*) denotes a statistically significant association (p ≤ 0.05). Statistical significance was interpreted based on the FDR-adjusted p-values.

Supplementary Table 5. Female veterinarians' perceptions of their emotional awareness and stress management skills according to professional experience (n = 152).

Statements (n=11)	Professional experience										
	0-5 years (n=66)	6-10 years (n=32)	11-20 years (n=39)	> 20 years (n=15)	Pearson's chi-square (χ2) test			Cramer's V Value	Kruskal Wallis		
	Mean ± SD				χ^2 (12)	p-value	FDR-adjusted p		H (3)	p-value	FDR-adjusted p
I am aware of my feelings and emotions as they are happening.	4.00 ± 0.72	4.06 ± 0.84	3.87 ± 0.86	4.40 ± 0.51	11.414	0.248	0.521	0.274	4.739	0.192	0.490
I can effectively manage my emotions and set boundaries when faced with challenging cases or unfair treatment.	3.00 ± 0.93	2.72 ± 0.92	2.95 ± 0.97	3.27 ± 1.03	13.610	0.326	0.596	0.299	4.535	0.209	0.495
I consciously take breaks and pauses during which I don't dwell on work and potential stressors.	2.79 ± 1.25	3.00 ± 1.14	3.10 ± 1.27	3.20 ± 1.08	8.588	0.738	0.869	0.238	2.371	0.499	0.734
I frequently carry work-related stress home, as I find myself preoccupied with it even after working hours.	3.80 ± 1.11	4.25 ± 0.92	3.95 ± 1.00	4.13 ± 1.06	6.457	0.891	0.933	0.206	4.258	0.235	0.507
I am able to identify stressors in my work environment.	3.95 ± 0.75	4.00 ± 0.72	3.74 ± 0.79	3.87 ± 0.74	6.474	0.692	0.856	0.206	2.668	0.446	0.699
I sometimes feel overwhelmed by the demands and responsibilities of my job as a veterinarian.	4.02 ± 1.03	3.38 ± 1.13	3.62 ±1.14	3.33 ± 0.62	25.622	***<0.05****	0.099	0.411	13.683	***<0.05****	***<0.05****
I have close personal relationship to a partner/friends/family/colleagues.	4.47 ± 0.88	4.25 ± 0.95	4.49 ± 0.89	4.33 ± 0.82	7.147	0.848	0.905	0.217	2.846	0.416	0.660
It is easy for me to talk about my emotions.	3.36 ± 1.06	3.41 ± 0.98	3.67 ± 1.22	3.93 ± 1.03	18.171	0.111	0.386	0.346	5.035	0.169	0.455
I am aware of my emotional comfort zone, i.e. I know how to experience and express emotions in a way they are comfortable for me.	3.06 ± 0.93	3.22 ± 0.94	3.41 ± 0.94	3.73 ± 1.10	17.835	0.121	0.407	0.343	6.796	0.079	0.302
I feel that my workload is manageable.	2.79 ± 1.05	2.97 ± 1.06	2.90 ± 1.17	3.60 ± 0.91	10.698	0.555	0.760	0.265	7.003	0.072	0.290
I often feel stress or experience burnout symptoms (e.g., emotional exhaustion, depersonalization, reduced personal accomplishment).	3.45 ± 1.19	3.00 ± 1.22	3.10 ± 1.29	2.73 ± 0.96	10.979	0.531	0.747	0.269	6.712	0.082	0.310

An asterisk (*) denotes a statistically significant association (p ≤ 0.05). Statistical significance was interpreted based on the FDR-adjusted p-values.

Supplementary Table 6. Female veterinarians' perceptions of their emotional awareness and stress management skills according to frequency of on-call shifts (n = 152).

Statements (n=11)	On-call shifts											
	No (n=48)	1x/month (n=34)	2-3x/ month (n=20)	1x/week (n=19)	>1/week (n=31)	Pearson's chi-square ($\chi 2$) test			Cramer's V Value	Kruskal Wallis		
	Mean ± SD					χ^2 (16)	p-value	FDR-adjusted p		H (4)	p-value	FDR-adjusted p
I am aware of my feelings and emotions as they are happening.	3.98 ± 0.89	4.15 ± 0.61	4.10 ± 0.91	3.95 ± 0.71	3.94 ± 0.73	11.201	0.512	0.739	0.271	2.169	0.705	0.861
I can effectively manage my emotions and set boundaries when faced with challenging cases or unfair treatment.	2.75 ± 1.00	3.38 ± 0.78	2.75 ± 0.97	2.84 ± 0.90	3.00 ± 0.97	17.231	0.371	0.625	0.337	11.189	***<0.05****	0.152
I consciously take breaks and pauses during which I don't dwell on work and potential stressors.	2.85 ± 1.20	3.35 ± 1.15	2.85 ± 1.35	2.74 ± 1.28	2.87 ± 1.15	12.516	0.708	0.861	0.287	4.648	0.325	0.596
I frequently carry work-related stress home, as I find myself preoccupied with it even after working hours.	4.08 ± 0.99	3.59 ± 1.02	4.25 ± 1.02	3.95 ± 1.18	4.03 ± 1.05	18.672	0.286	0.560	0.350	7.770	0.100	0.367
I am able to identify stressors in my work environment.	3.92 ± 0.82	3.88 ± 0.77	3.85 ± 0.81	3.89 ± 0.81	3.94 ± 0.57	14.675	0.260	0.534	0.311	0.189	0.996	0.996
I sometimes feel overwhelmed by the demands and responsibilities of my job as a veterinarian.	3.67 ± 1.14	3.82 ± 1.00	3.85 ± 1.04	3.89 ± 0.74	3.45 ± 1.26	17.722	0.340	0.610	0.341	1.664	0.797	0.892
I have close personal relationship to a partner/friends/family/colleagues.	4.38 ± 0.98	4.29 ± 0.94	4.65 ± 0.49	4.68 ± 0.48	4.29 ± 1.04	8.271	0.940	0.968	0.233	2.875	0.579	0.783
It is easy for me to talk about my emotions.	3.63 ± 1.04	3.47 ± 1.29	3.60 ± 1.10	3.47 ± 0.96	3.32 ± 1.05	11.571	0.773	0.891	0.276	1.575	0.813	0.892
I am aware of my emotional comfort zone, i.e. I know how to experience and express emotions in a way they are comfortable for me.	3.25 ± 0.96	3.29 ± 0.91	3.20 ± 1.20	3.32 ± 1.00	3.19 ± 0.91	10.962	0.812	0.892	0.269	0.384	0.984	0.991
I feel that my workload is manageable.	3.13 ± 1.04	3.12 ± 0.77	2.90 ± 1.12	2.63 ± 1.12	2.65 ± 1.33	28.021	***<0.05****	0.170	0.429	6.603	0.158	0.446
I often feel stress or experience burnout symptoms (e.g., emotional exhaustion, depersonalization, reduced personal accomplishment).	3.17 ± 1.31	3.00 ± 1.04	3.55 ± 1.10	3.53 ± 1.17	3.03 ± 1.33	21.264	0.169	0.455	0.374	4.631	0.327	0.596

An asterisk (*) denotes a statistically significant association ($p \leq 0.05$). Statistical significance was interpreted based on the FDR-adjusted p-values.

Supplementary Table 7. Female veterinarians' perceptions of their emotional awareness and stress management skills according to work-life balance (n = 152).

Statements (n=11)	Work-life balance									
	Excellent of Good (n=51)	Fair (n=67)	Poor or Very poor (n=34)	Pearson's chi-square ($\chi2$) test			Cramer's V Value	Kruskal-Wallis		
	Mean ± SD			$\chi^2(8)$	p-value	FDR-adjusted p		H (2)	p-value	FDR-adjusted p
I am aware of my feelings and emotions as they are happening.	4.14 ± 0.63	3.96 ± 0.86	3.97 ± 0.80	15.340	<0.05*	0.126	0.318	1.297	0.523	0.746
I can effectively manage my emotions and set boundaries when faced with challenging cases or unfair treatment.	3.20 ± 0.92	2.93 ± 0.91	2.65 ± 1.01	8.794	0.36	0.617	0.241	7.015	<0.05*	0.167
I consciously take breaks and pauses during which I don't dwell on work and potential stressors.	3.63 ± 1.31	2.82 ± 1.15	2.21 ± 0.91	35.144	<0.0001*	<0.01*	0.481	29.172	<0.0001*	<0.0001*
I frequently carry work-related stress home, as I find myself preoccupied with it even after working hours.	3.76 ± 1.03	3.84 ± 1.12	4.53 ± 0.66	17.049	<0.05*	0.167	0.335	13.124	<0.001*	<0.05*
I am able to identify stressors in my work environment.	3.80 ± 0.83	4.00 ± 0.65	3.85 ± 0.82	8.432	0.208	0.495	0.236	1.498	0.473	0.716
I sometimes feel overwhelmed by the demands and responsibilities of my job as a veterinarian.	3.67 ± 1.13	3.55 ± 1.05	4.09 ± 1.00	10.932	0.206	0.495	0.268	6.615	<0.05*	0.192
I have close personal relationship to a partner/friends/family/colleagues.	4.53 ± 0.81	4.48 ± 0.73	4.12 ± 1.20	10.770	0.215	0.495	0.266	3.041	0.219	0.499
It is easy for me to talk about my emotions.	3.82 ± 1.09	3.43 ± 1.06	3.18 ± 1.06	12.322	0.137	0.413	0.285	8.924	<0.05*	0.098
I am aware of my emotional comfort zone, i.e. I know how to experience and express emotions in a way they are comfortable for me.	3.57 ± 0.92	3.13 ± 0.95	3.00 ± 0.95	11.160	0.193	0.490	0.271	8.56	<0.05*	0.104
I feel that my workload is manageable.	3.76 ± 0.82	2.81 ± 0.94	1.94 ± 0.69	73.359	<0.0001*	<0.0001*	0.695	61.417	<0.0001*	<0.0001*
I often feel stress or experience burnout symptoms (e.g., emotional exhaustion, depersonalization, reduced personal accomplishment).	2.55 ± 1.12	3.22 ± 1.06	4.12 ± 1.07	51.543	<0.0001*	<0.0001*	0.582	34.591	<0.0001*	<0.0001*

An asterisk (*) denotes a statistically significant association ($p \leq 0.05$). Statistical significance was interpreted based on the FDR-adjusted p-values.

Supplementary Table 8. Female veterinarians' perceptions of their emotional awareness and stress management skills according to satisfaction with support for professional development and continuing education (n = 152).

Statements (n=11)	Satisfaction with the support provided for professional development and continuing education									
	(Very) satisfied (n=77)	Neutral (n=33)	(Very) dissatisfied (n=42)	Pearson's chi-square (χ2) test			Cramer's V Value	Kruskal-Wallis		
	Mean ± SD			$\chi^2(8)$	p-value	FDR-adjusted p		H (2)	p-value	FDR-adjusted p
I am aware of my feelings and emotions as they are happening.	4.01 ± 0.75	4.15 ± 0.67	3.93 ± 0.89	4.294	0.637	0.835	0.168	0.892	0.64	0.835
I can effectively manage my emotions and set boundaries when faced with challenging cases or unfair treatment.	2.99 ± 0.90	2.97 ± 1.05	2.88 ± 0.99	4.114	0.847	0.905	0.165	0.468	0.791	0.892
I consciously take breaks and pauses during which I don't dwell on work and potential stressors.	3.01 ± 1.25	3.09 ± 1.18	2.74 ± 1.17	7.728	0.461	0.707	0.225	2.294	0.318	0.592
I frequently carry work-related stress home, as I find myself preoccupied with it even after working hours.	3.73 ± 1.11	4.30 ± 0.77	4.14 ± 1.03	12.384	0.135	0.413	0.285	7.937	***<0.05****	0.127
I am able to identify stressors in my work environment.	3.94 ± 0.75	3.76 ± 0.79	3.95 ± 0.73	4.050	0.670	0.852	0.163	1.837	0.399	0.644
I sometimes feel overwhelmed by the demands and responsibilities of my job as a veterinarian.	3.57 ± 1.16	4.00 ± 0.83	3.74 ± 1.04	10.694	0.220	0.499	0.265	2.668	0.263	0.534
I have close personal relationship to a partner/friends/family/colleagues.	4.49 ± 0.68	4.36 ± 0.96	4.31 ± 1.14	10.879	0.209	0.495	0.268	0.008	0.996	0.996
It is easy for me to talk about my emotions.	3.56 ± 1.06	3.52 ± 1.12	3.40 ± 1.15	5.873	0.661	0.847	0.197	0.627	0.731	0.867
I am aware of my emotional comfort zone, i.e. I know how to experience and express emotions in a way they are comfortable for me.	3.25 ± 0.99	3.33 ± 0.82	3.19 ± 1.04	7.339	0.500	0.734	0.220	0.384	0.825	0.897
I feel that my workload is manageable.	3.17 ± 1.08	2.94 ± 1.00	2.50 ± 1.04	16.347	***<0.05****	0.192	0.328	10.846	***<0.05****	***<0.05****
I often feel stress or experience burnout symptoms (e.g., emotional exhaustion, depersonalization, reduced personal accomplishment).	2.81 ± 1.15	3.33 ± 1.05	3.81 ± 1.22	28.710	***<0.0001****	***<0.01****	0.435	19.755	***<0.0001****	***<0.01****

An asterisk (*) denotes a statistically significant association (p ≤ 0.05). Statistical significance was interpreted based on the FDR-adjusted p-values.

Supplementary Table 9. Female veterinarians' responses to workload, client-related stressors, collegial support, and professional self-efficacy according to age group (n = 152).

Statements (n=7)	Age group									
	23-34 years (n=66)	35-44 years (n=56)	>45 years (n=30)	Pearson's chi-square (χ2) test			Cramer's V Value	Kruskal Wallis		
	Mean ± SD			χ^2 (8)	p-value	FDR-adjusted p		H (2)	p-value	FDR-adjusted p
I cancel leisure activities when faced with an unusually high workload.	3.70 ± 1.29	3.64 ± 1.17	4.20 ± 0.76	8.253	0.409	0.655	0.233	3.988	0.136	0.413
When a client rises his voice against me it affects me in a long term.	4.05 ± 1.10	3.88 ± 1.15	3.97 ± 1.07	3.682	0.885	0.930	0.156	0.742	0.690	0.856
I can discuss difficult cases with other (more experienced) colleagues.	4.15 ± 0.85	4.20 ± 0.88	3.63 ± 1.35	11.873	0.157	0.446	0.279	3.625	0.163	0.454
I take part in training courses to be up to date and feel more self-confident.	4.06 ± 0.89	4.38 ± 0.70	4.23 ± 0.94	6.325	0.388	0.634	0.204	3.696	0.185	0.446
My team works on optimizing processes to work more efficient.	2.88 ± 1.14	3.38 ± 1.15	3.60 ± 1.40	19.789	***<0.05****	0.097	0.361	9.653	***<0.01****	0.079
I perceive empathy or understanding from the client's side towards me in case adverse effects happen.	2.94 ± 0.88	3.09 ± 1.00	3.17 ± 1.21	13.273	0.103	0.369	0.296	1.317	0.518	0.742
I perceive empathy or understanding from colleagues towards me in case adverse effects happen.	3.77 ± 1.01	3.79 ± 0.99	3.90 ± 1.06	6.486	0.593	0.799	0.207	0.462	0.794	0.892

An asterisk (*) denotes a statistically significant association ($p \leq 0.05$). Statistical significance was interpreted based on the FDR-adjusted p-values.

Supplementary Table 10. Female veterinarians' responses to workload, client-related stressors, collegial support, and professional self-efficacy according to marital status (n = 152).

Statements (n=7)	Marital status									
	Unmarried (n=96)	Married (n=56)	Pearson's chi-square (χ2) test			Cramer's V Value	Mann-Whitney			
	Mean ± SD		χ^2 (4)	p-value	FDR-adjusted p		U	Z	p-value	FDR-adjusted p
I cancel leisure activities when faced with an unusually high workload.	3.70 ± 1.27	3.91 ± 0.96	7.043	0.134	0.413	0.215	2561.5	-0.507	0.612	0.815
When a client rises his voice against me it affects me in a long term.	3.99 ± 1.09	3.93 ± 1.14	2.034	0.729	0.867	0.116	2627.5	-0.244	0.807	0.892
I can discuss difficult cases with other (more experienced) colleagues.	4.10 ± 0.95	4.00 ± 1.08	1.563	0.815	0.892	0.101	2586.5	-0.414	0.679	0.852
I take part in training courses to be up to date and feel more self-confident.	4.21 ± 0.82	4.21 0.89	0.804	0.849	0.905	0.073	2631.0	-0.235	0.814	0.892
My team works on optimizing processes to work more efficient.	3.04 ± 1.22	3.48 ± 1.21	7.133	0.129	0.413	0.217	2117.5	-2.237	***<0.05****	0.155
I perceive empathy or understanding from the client's side towards me in case adverse effects happen.	2.97 ± 0.96	3.16 ±1.04	2.388	0.665	0.848	0.125	2400.5	-1.162	0.245	0.521
I perceive empathy or understanding from colleagues towards me in case adverse effects happen.	3.70 ± 1.00	3.98 ± 1.00	6.502	0.165	0.455	0.207	2260.0	-1.709	0.087	0.327

An asterisk (*) denotes a statistically significant association ($p \leq 0.05$). Statistical significance was interpreted based on the FDR-adjusted p-values.

Supplementary Table 11. Female veterinarians' responses to workload, client-related stressors, collegial support, and professional self-efficacy according to weekly working hours (n = 152).

Statements (n=7)	Weekly working hours									
	≤ 40 h (n=95)	> 40 h (n=57)	Pearson's chi-square (χ2) test			Cramer's V Value	Mann- Whitney			
	Mean ± SD		χ^2 (4)	p-value	FDR-adjusted p		U	Z	p-value	FDR-adjusted p
I cancel leisure activities when faced with an unusually high workload.	3.64 ± 1.19	4.00 ± 1.10	4.500	0.343	0.610	0.172	2223.0	-1.935	0.053	0.238
When a client rises his voice against me it affects me in a long term.	4.03 ± 1.14	3.86 ± 1.04	5.832	0.212	0.495	0.196	2397.0	-1.249	0.212	0.495
I can discuss difficult cases with other (more experienced) colleagues.	4.00 ± 1.07	4.18 ± 0.85	3.183	0.528	0.747	0.145	2532.5	-0.711	0.477	0.716
I take part in training courses to be up to date and feel more self-confident.	4.22 ± 0.90	4.19 ± 0.74	5.954	0.114	0.394	0.198	2543.0	-0.675	0.500	0.734
My team works on optimizing processes to work more efficient.	3.20 ± 1.22	3.21 ± 1.26	0.884	0.925	0.959	0.076	2696.0	-0.045	0.964	0.986
I perceive empathy or understanding from the client's side towards me in case adverse effects happen.	3.11 ± 0.95	2.93 ± 1.05	2.326	0.676	0.852	0.124	2463.0	-0.985	0.325	0.596
I perceive empathy or understanding from colleagues towards me in case adverse effects happen.	3.82 ± 0.99	3.77 ± 1.04	0.207	0.955	0.996	0.037	2648.5	-0.235	0.814	0.892

Supplementary Table 12. Female veterinarians' responses to workload, client-related stressors, collegial support, and professional self-efficacy according to professional position (n = 152).

Statements (n=7)	Position									
	Employed (n=120)	Employer (n=32)	Pearson's chi-square (χ2) test			Cramer's V Value	Mann- Whitney			
	Mean ± SD		χ^2 (4)	p-value	FDR-adjusted p		U	Z	p-value	FDR-adjusted p
I cancel leisure activities when faced with an unusually high workload.	3.73 ± 1.20	3.97 ± 1.03	2.126	0.713	0.861	0.118	1735.5	-0.875	0.382	0.629
When a client rises his voice against me it affects me in a long term.	4.01 ± 1.10	3.81 ± 1.15	1.629	0.804	0.892	0.104	1731.0	-0.903	0.366	0.625
I can discuss difficult cases with other (more experienced) colleagues.	4.12 ± 0.93	3.88 ± 1.10	5.509	0.239	0.513	0.190	1670.0	-1.205	0.288	0.506
I take part in training courses to be up to date and feel more self-confident.	4.15 ± 0.88	4.44 ± 0.67	3.245	0.355	0.613	0.146	1601.0	-1.554	0.120	0.407
My team works on optimizing processes to work more efficient.	3.03 ± 1.21	3.88 ± 1.07	12.313	***<0.01****	0.092	0.296	1166.5	-3.495	***<0.0001****	***<0.01****
I perceive empathy or understanding from the client's side towards me in case adverse effects happen.	2.92 ±0.89	3.50 ± 1.19	25.573	***<0.0001****	***<0.01****	0.394	1318.0	-2.879	***<0.05****	0.045
I perceive empathy or understanding from colleagues towards me in case adverse effects happen.	3.82 ± 0.97	3.75 ± 1.14	5.807	0.214	0.495	0.195	1881.0	-0.184	0.854	0.908

An asterisk (*) denotes a statistically significant association (p ≤ 0.05). Statistical significance was interpreted based on the FDR-adjusted p-values.

Supplementary Table 13. Female veterinarians' responses to workload, client interactions, collegial support, and professional confidence according to professional experience (n = 152).

Statements (n=7)	Professional experience										
	0-5 years (n=66)	6-10 years (n=32)	11-20 years (n=39)	> 20 years (n=15)	Pearson's chi-square (χ2) test			Cramer's V Value	Kruskal Wallis		
	Mean ± SD				χ^2 (12)	p-value	FDR-adjusted p		H (3)	p-value	FDR-adjusted p
I cancel leisure activities when faced with an unusually high workload.	0.76 ± 1.25	3.81 ± 1.23	3.64 ± 1.09	4.13 ± 0.83	7.570	0.818	0.892	0.223	2.183	0.535	0.747
When a client rises his voice against me it affects me in a long term.	3.95 ± 1.13	4.16 ± 1.05	3.95 ± 1.12	3.67 ± 1.11	7.057	0.854	0.908	0.215	2.310	0.511	0.739
I can discuss difficult cases with other (more experienced) colleagues.	4.30 ± 0.82	3.88 ± 1.07	3.74 ± 1.07	4.27 ± 1.10	15.640	0.208	0.495	0.321	10.763	***<0.05****	0.100
I take part in training courses to be up to date and feel more self-confident.	4.06 ± 0.86	4.50 ± 0.76	4.21 ± 0.80	4.27 ± 0.96	8.872	0.449	0.699	0.242	7.142	0.068	0.279
My team works on optimizing processes to work more efficient.	2.97 ± 1.11	3.16 ± 1.25	3.41 ± 1.31	3.80 ± 1.32	22.445	***<0.05****	0.175	0.384	8.055	***<0.05****	0.211
I perceive empathy or understanding from the client's side towards me in case adverse effects happen.	2.97 ± 0.84	2.91 ± 1.03	2.97 ± 1.11	3.80 ± 0.94	21.147	***<0.05****	0.223	0.373	8.959	***<0.05****	0.167
I perceive empathy or understanding from colleagues towards me in case adverse effects happen.	3.79 ± 1.03	3.88 ± 0.98	3.67 ± 1.01	4.07 ± 0.96	7.688	0.809	0.892	0.225	2.091	0.554	0.760

An asterisk (*) denotes a statistically significant association ($p \leq 0.05$). Statistical significance was interpreted based on the FDR-adjusted p-values.

Supplementary Table 14. Female veterinarians' responses to workload, client interactions, collegial support, and professional confidence according to frequency of on-call shifts (n = 152).

Statements (n=7)	On-call shifts											
	No (n=48)	1x/ month (n=34)	2-3x/ month (n=20)	1x/week (n=19)	>1/week (n=31)	Pearson's chi-square (χ2) test			Cramer's V Value	Kruskal Wallis		
	Mean ± SD					χ^2 (16)	p-value	FDR-adjusted p		H (4)	p-value	FDR-adjusted p
I cancel leisure activities when faced with an unusually high workload.	3.73 ± 1.28	3.65 ± 1.07	3.75 ± 1.12	3.53 ± 1.17	4.16 ± 1.10	19.360	0.250	0.523	0.357	6.592	0.159	0.446
When a client rises his voice against me it affects me in a long term.	4.10 ± 1.04	3.68 ± 1.22	3.75 ± 1.12	4.11 ± 1.10	4.13 ± 1.06	10.879	0.817	0.892	0.268	4.605	0.33	0.598
I can discuss difficult cases with other (more experienced) colleagues.	4.00 ± 1.01	4.12 ± 0.95	4.15 ± 0.88	4.05 ± 0.97	4.06 ± 1.15	7.036	0.973	0.987	0.215	0.557	0.968	0.986
I take part in training courses to be up to date and feel more self-confident.	4.23 ± 0.88	4.29 ± 0.91	3.90 ± 0.85	4.26 ± 0.81	4.26 ± 0.73	16.588	0.166	0.455	0.330	4.006	0.405	0.651
My team works on optimizing processes to work more efficient.	3.06 ± 1.23	3.35 ± 1.04	3.35 ± 1.39	3.16 ± 1.39	3.19 ± 1.28	8.955	0.915	0.952	0.243	1.414	0.842	0.905
I perceive empathy or understanding from the client's side towards me in case adverse effects happen.	2.90 ± 0.99	3.15 ± 0.96	3.00 ± 0.97	2.79 ± 0.92	3.32 ± 1.05	10.864	0.818	0.892	0.267	5.873	0.209	0.495
I perceive empathy or understanding from colleagues towards me in case adverse effects happen.	3.69 ± 0.95	3.82 ± 1.06	4.25 ± 0.85	3.68 ± 1.11	3.74 ± 1.03	16.597	0.412	0.657	0.330	5.041	0.283	0.560

Supplementary Table 15. Female veterinarians' responses to workload, client interactions, collegial support, and professional confidence according to work-life balance (n = 152).

Statements (n=7)	Work-life balance									
	Excellent of Good (n=51)	Fair (n=67)	Poor or Very poor (n=34)	Pearson's chi-square (χ2) test			Cramer's V Value	Kruskal-Wallis		
	Mean ± SD			χ^2 (8)	p-value	FDR-adjusted p		H (2)	p-value	FDR-adjusted p
I cancel leisure activities when faced with an unusually high workload.	3.31 ± 1.12	3.90 ± 1.16	4.24 ± 1.05	28.101	***<0.0001****	***<0.01****	0.430	17.572	***<0.0001****	***<0.01****
When a client rises his voice against me it affects me in a long term.	3.82 ± 1.11	3.91 ± 1.16	4.29 ± 0.94	5.148	0.742	0.871	0.184	4.053	0.132	0.413
I can discuss difficult cases with other (more experienced) colleagues.	4.39 ± 0.70	4.03 ± 0.94	3.65 ± 1.30	15.576	***<0.05****	0.223	0.32	8.506	***<0.05****	0.104
I take part in training courses to be up to date and feel more self-confident.	4.24 ± 0.89	4.25 ± 0.79	4.09 ± 0.90	5.616	0.468	0.713	0.192	0.871	0.647	0.835
My team works on optimizing processes to work more efficient.	3.53 ± 1.19	3.28 ± 1.17	2.56 ± 1.21	15.923	***<0.05****	0.209	0.324	12.653	***<0.01****	***<0.05****
I perceive empathy or understanding from the client's side towards me in case adverse effects happen.	3.39 ± 0.87	3.12 ± 0.90	2.35 ± 1.01	29.261	***<0.0001****	0.006	0.439	20.410	***<0.0001****	***<0.01****
I perceive empathy or understanding from colleagues towards me in case adverse effects happen.	4.18 ± 0.77	3.75 ± 0.99	3.35 ± 1.15	22.793	***<0.01****	***<0.05****	0.387	12.309	***<0.01****	***<0.05****

An asterisk (*) denotes a statistically significant association ($p \leq 0.05$). Statistical significance was interpreted based on the FDR-adjusted p-values.

Supplementary Table 16. Female veterinarians' responses to workload, client interactions, collegial support, and professional confidence according to satisfaction with support for professional development and continuing education (n = 152).

Statements (n=7)	Satisfaction with the support provided for professional development and continuing education									
	(Very) satisfied (n=77)	Neutral (n=33)	(Very) dissatisfied (n=42)	Pearson's chi-square (χ2) test			Cramer's V Value	Kruskal-Wallis		
	Mean ± SD			χ^2 (8)	p-value	FDR-adjusted p		H (2)	p-value	FDR-adjusted p
I cancel leisure activities when faced with an unusually high workload.	3.56 ± 1.09	3.76 ± 1.28	4.19 ± 1.13	22.420	***<0.01****	0.046	0.384	11.829	***<0.01****	***<0.05****
When a client rises his voice against me it affects me in a long term.	3.83 ± 1.16	4.36 ± 0.90	3.90 ± 1.10	7.056	0.531	0.747	0.215	5.517	0.063	0.271
I can discuss difficult cases with other (more experienced) colleagues.	4.38 ± 0.73	3.85 ± 1.03	3.67 ± 1.20	24.189	***<0.01****	***<0.05****	0.399	13.699	***<0.01****	***<0.05****
I take part in training courses to be up to date and feel more self-confident.	4.47 ± 0.68	3.94 ± 0.86	3.95 ± 0.96	17.090	***<0.01****	0.086	0.335	13.722	***<0.001****	***<0.05****
My team works on optimizing processes to work more efficient.	3.62 ± 1.06	3.33 ± 1.19	2.33 ± 1.12	33.417	***<0.0001****	***<0.01****	0.469	29.436	***<0.0001****	***<0.0001****
I perceive empathy or understanding from the client's side towards me in case adverse effects happen.	3.09 ± 0.96	3.27 ± 0.84	2.76 ± 1.10	15.939	***<0.05****	0.209	0.324	5.350	0.069	0.282
I perceive empathy or understanding from colleagues towards me in case adverse effects happen.	4.08 ± 0.89	3.85 ± 0.94	3.26 ± 1.06	21.661	***<0.01****	0.058	0.377	17.069	***<0.0001****	***<0.01****

An asterisk (*) denotes a statistically significant association (p ≤ 0.05). Statistical significance was interpreted based on the FDR-adjusted p-values.

Supplementary Table 17. Female veterinarians' emotional and professional responses to mistakes or adverse events according to age group (n = 152).

Statements (n=6)	Age group									
	23-34 years (n=66)	35-44 years (n=56)	>45 years (n=30)	Pearson's chi-square (χ2) test			Cramer's V Value	Kruskal Wallis		
	Mean ± SD			χ^2 (8)	p-value	FDR-adjusted p		H (2)	p-value	FDR-adjusted p
I am afraid of making the same mistake again and avoid similar cases if possible.	3.26 ± 0.97	2.96 ± 1.14	2.90 ± 1.03	9.516	0.301	0.574	0.25	3.219	0.200	0.495
I feel more insecure and cautious when handling a similar case.	3.62 ± 0.92	3.63 ± 1.04	3.57 ± 1.07	8.606	0.377	0.627	0.238	0.154	0.926	0.959
I immediately try to change protocols or procedures regarding similar cases.	3.39 ± 1.08	3.64 ± 0.96	3.80 ± 0.93	7.404	0.494	0.732	0.221	3.573	0.168	0.455
I actively seek ways to improve my skills and knowledge to prevent it from happening again.	4.26 ± 0.81	4.45 ± 0.74	4.20 ± 0.71	7.294	0.295	0.568	0.219	3.454	0.178	0.474
I acknowledge that mistakes can happen and do not let it affect my confidence significantly.	2.86 ± 0.98	3.00 ± 1.11	3.03 ± 1.27	6.350	0.608	0.811	0.204	0.692	0.707	0.861
I remain confident in my knowledge and abilities despite the mistake.	2.92 ± 1.00	3.34 ± 1.16	3.27 ± 1.05	12.261	0.140	0.417	0.284	5.592	0.061	0.266

Supplementary Table 18. Female veterinarians' emotional and professional responses to mistakes or adverse events according to marital status (n = 152).

Statements (n=6)	Marital status									
	Unmarried (n=96)	Married (n=56)	Pearson's chi-square (χ2) test			Cramer's V Value	Mann-Whitney			
	Mean ± SD		χ^2 (4)	p-value	FDR-adjusted p		U	Z	p-value	FDR-adjusted p
I am afraid of making the same mistake again and avoid similar cases if possible.	3.14 ± 1.04	2.98 ±1.07	1.245	0.871	0.919	0.090	2467.0	-0.877	0.380	0.629
I feel more insecure and cautious when handling a similar case.	3.65 ± 0.98	3.55 ± 1.01	1.282	0.864	0.914	0.092	2540.5	-0.596	0.551	0.760
I immediately try to change protocols or procedures regarding similar cases.	3.58 ± 1.01	3.54 ± 1.03	0.422	0.981	0.991	0.053	2624.5	-0.255	0.799	0.892
I actively seek ways to improve my skills and knowledge to prevent it from happening again.	4.29 ± 0.79	4.36 ± 0.72	0.527	0.913	0.952	0.059	2600.0	-0.369	0.712	0.861
I acknowledge that mistakes can happen and do not let it affect my confidence significantly.	2.94 ± 1.09	2.96 ± 1.08	1.571	0.814	0.892	0.102	2674.5	-0.053	0.957	0.985
I remain confident in my knowledge and abilities despite the mistake.	3.08 ±1.09	3.25 ± 1.07	8.840	0.065	0.272	0.241	2422.5	-1.054	0.292	0.568

Supplementary Table 19. Female veterinarians' emotional and professional responses to mistakes or adverse events according to weekly working hours (n = 152).

Statements (n=6)	Weekly working hours									
	≤ 40 h (n=95)	> 40 h (n=57)	Pearson's chi-square (χ2) test			Cramer's V Value	Mann- Whitney			
	Mean ± SD		χ^2 (4)	p-value	FDR-adjusted p		U	Z	p-value	FDR-adjusted p
I am afraid of making the same mistake again and avoid similar cases if possible.	3.02 ± 1.04	3.18 ± 1.07	2.321	0.677	0.852	0.124	2517.5	-0.751	0.452	0.702
I feel more insecure and cautious when handling a similar case.	3.65 ± 0.94	3.54 ± 1.07	5.827	0.212	0.495	0.196	2622.5	-0.342	0.732	0.867
I immediately try to change protocols or procedures regarding similar cases.	3.48 ± 1.03	3.70 ± 0.98	2.140	0.710	0.861	0.119	2376.5	-1.325	0.185	0.486
I actively seek ways to improve my skills and knowledge to prevent it from happening again.	4.25 ± 0.81	4.42 ± 0.68	3.093	0.377	0.627	0.143	2437.5	-1.127	0.260	0.534
I acknowledge that mistakes can happen and do not let it affect my confidence significantly.	3.00 ± 1.01	2.86 ± 1.60	4.174	0.383	0.629	0.166	2528.0	-0.708	0.479	0.716
I remain confident in my knowledge and abilities despite the mistake.	3.17 ± 1.07	3.11 ± 1.11	2.299	0.681	0.852	0.123	2630.0	-0.306	0.759	0.880

Supplementary Table 20. Female veterinarians' emotional and professional responses to mistakes or adverse events according to professional position (n = 152).

Statements (n=6)	Position									
	Employed (n=120)	Employer (n=32)	Pearson's chi-square (χ2) test			Cramer's V Value	Mann- Whitney			
	Mean ± SD		χ² (4)	p-value	FDR-adjusted p		U	Z	p-value	FDR-adjusted p
I am afraid of making the same mistake again and avoid similar cases if possible.	3.13 ± 1.05	2.88 ± 1.07	1.951	0.745	0.872	0.113	1660.0	-1.221	0.222	0.502
I feel more insecure and cautious when handling a similar case.	3.67 ± 1.01	3.41 ± 0.91	5.020	0.285	0.560	0.182	1590.5	-1.576	0.115	0.397
I immediately try to change protocols or procedures regarding similar cases.	3.45 ± 1.05	4.00 ± 0.72	9.292	0.054	0.239	0.247	1355.5	-2.683	***<0.01****	0.074
I actively seek ways to improve my skills and knowledge to prevent it from happening again.	4.26 ± 0.79	4.53 ± 0.62	3.271	0.352	0.613	0.147	1574.0	-1.715	0.086	0.327
I acknowledge that mistakes can happen and do not let it affect my confidence significantly.	2.83 ± 0.98	3.38 ± 1.34	22.127	***<0.0001****	***<0.01****	0.382	1415.0	-2.364	***<0.05****	0.127
I remain confident in my knowledge and abilities despite the mistake.	2.98 ± 1.04	3.75 ± 1.05	18.003	***<0.001****	***<0.05****	0.344	1160.5	-3.567	***<0.0001****	***<0.01****

An asterisk (*) denotes a statistically significant association (p ≤ 0.05). Statistical significance was interpreted based on the FDR-adjusted p-values.

Supplementary Table 21. Female veterinarians' emotional and professional responses to mistakes or adverse events according to professional experience (n = 152).

Statements (n=6)	Professional experience										
	0-5 years (n=66)	6-10 years (n=32)	11-20 years (n=39)	> 20 years (n=15)	Pearson's chi-square ($\chi 2$) test			Cramer's V Value	Kruskal Wallis		
	Mean ± SD				χ^2 (12)	p-value	FDR-adjusted p		H (3)	p-value	FDR-adjusted p
I am afraid of making the same mistake again and avoid similar cases if possible.	3.27 ± 1.05	2.94 ± 0.98	3.05 ± 1.10	2.60 ± 0.99	10.951	0.533	0.747	0.268	5.641	0.130	0.413
I feel more insecure and cautious when handling a similar case.	3.62 ± 0.96	3.91 ± 0.82	3.46 ± 1.10	3.33 ± 1.11	13.179	0.356	0.613	0.294	4.869	0.182	0.478
I immediately try to change protocols or procedures regarding similar cases.	3.44 ± 1.04	3.78 ± 1.01	3.44 ± 1.05	4.00 ± 0.66	10.687	0.556	0.760	0.256	6.038	0.110	0.386
I actively seek ways to improve my skills and knowledge to prevent it from happening again.	4.33 ± 0.75	4.59 ± 0.71	4.05 ± 0.73	4.33 ± 0.72	13.706	0.133	0.413	0.300	10.797	***<0.05****	0.100
I acknowledge that mistakes can happen and do not let it affect my confidence significantly.	2.80 ± 0.98	3.03 ± 1.03	2.79 ± 1.15	3.80 ± 1.15	20.820	0.053	0.238	0.370	10.921	***<0.05****	0.099
I remain confident in my knowledge and abilities despite the mistake.	2.80 ± 1.00	3.34 ± 1.13	3.28 ± 1.08	3.87 ± 0.92	24.018	***<0.05****	0.132	0.398	15.555	***<0.001****	***<0.05****

An asterisk (*) denotes a statistically significant association ($p \leq 0.05$). Statistical significance was interpreted based on the FDR-adjusted p-values.

Supplementary Table 22. Female veterinarians' emotional and professional responses to mistakes or adverse events according to frequency of on-call shifts (n = 152).

Statements (n=6)	On-call shifts											
	No (n=48)	1x/ month (n=34)	2-3x/ month (n=20)	1x/week (n=19)	>1/week (n=31)	Pearson's chi-square (χ2) test			Cramer's V Value	Kruskal Wallis		
	Mean ± SD					χ^2 (16)	p-value	FDR-adjusted p		H (4)	p-value	FDR-adjusted p
I am afraid of making the same mistake again and avoid similar cases if possible.	3.17 ± 0.93	2.82 ± 1.19	3.00 ± 1.21	3.05 ± 1.08	3.29 ± 0.94	25.365	0.064	0.271	0.409	3.480	0.481	0.716
I feel more insecure and cautious when handling a similar case.	3.85 ± 0.95	3.38 ± 0.95	3.60 ± 1.14	3.42 ± 1.12	3.61 ± 0.88	18.789	0.280	0.557	0.352	4.755	0.313	0.587
I immediately try to change protocols or procedures regarding similar cases.	3.54 ± 0.87	3.76 ± 0.99	3.70 ± 1.34	3.32 ± 1.00	3.45 ± 1.03	21.670	0.154	0.446	0.378	4.452	0.384	0.613
I actively seek ways to improve my skills and knowledge to prevent it from happening again.	4.35 ± 0.57	4.38 ± 0.82	4.50 ± 0.69	4.00 ± 1.00	4.26 ± 0.86	17.118	0.145	0.426	0.336	3.756	0.440	0.695
I acknowledge that mistakes can happen and do not let it affect my confidence significantly.	2.73 ± 1.09	2.97 ± 1.09	2.95 ± 1.23	2.84 ± 1.02	3.32 ± 0.98	19.521	0.243	0.517	0.358	7.022	0.135	0.413
I remain confident in my knowledge and abilities despite the mistake.	2.83 ± 1.16	3.18 ± 1.06	3.35 ± 0.99	3.05 ± 1.03	3.52 ± 1.00	19.771	0.231	0.506	0.361	7.610	0.107	0.380

Supplementary Table 23. Female veterinarians' emotional and professional responses to mistakes or adverse events according to work-life balance (n = 152).

Statements (n=6)	Work-life balance									
	Excellent of Good (n=51)	Fair (n=67)	Poor or Very poor (n=34)	Pearson's chi-square (χ2) test			Cramer's V Value	Kruskal-Wallis		
	Mean ± SD			χ² (8)	p-value	FDR-adjusted p		H (2)	p-value	FDR-adjusted p
I am afraid of making the same mistake again and avoid similar cases if possible.	2.90 ± 0.94	2.97 ± 1.10	3.56 ± 0.99	13.712	0.090	0.331	0.300	9.05	***<0.05****	0.097
I feel more insecure and cautious when handling a similar case.	3.63 ± 0.85	3.42 ± 1.03	3.97 ± 1.03	17.625	***<0.05****	0.152	0.341	7.828	***<0.05****	0.132
I immediately try to change protocols or procedures regarding similar cases.	3.69 ± 0.93	3.54 ± 1.02	3.44 ± 1.13	5.011	0.756	0.877	0.182	0.972	0.615	0.815
I actively seek ways to improve my skills and knowledge to prevent it from happening again.	4.41 ± 0.67	4.28 ± 0.87	4.24 ± 0.70	7.881	0.247	0.521	0.228	1.341	0.511	0.739
I acknowledge that mistakes can happen and do not let it affect my confidence significantly.	3.10 ± 0.96	3.09 ± 1.04	2.44 ± 1.21	32.715	***<0.0001****	***<0.01****	0.464	7.541	***<0.05****	0.147
I remain confident in my knowledge and abilities despite the mistake.	3.37 ± 1.02	3.18 ± 1.10	2.74 ± 1.05	8.613	0.376	0.627	0.238	7.027	***<0.05****	0.167

An asterisk (*) denotes a statistically significant association (p ≤ 0.05). Statistical significance was interpreted based on the FDR-adjusted p-values.

Supplementary Table 24. Female veterinarians' emotional and professional responses to mistakes or adverse events according to satisfaction with support for professional development and continuing education (n = 152).

Statements (n=6)	Satisfaction with the support provided for professional development and continuing education									
	(Very) satisfied (n=77)	Neutral (n=33)	(Very) dissatisfied (n=42)	Pearson's chi-square ($\chi 2$) test			Cramer's V Value	Kruskal-Wallis		
	Mean ± SD			χ^2 (8)	p-value	FDR-adjusted p		H (2)	p-value	FDR-adjusted p
I am afraid of making the same mistake again and avoid similar cases if possible.	2.91 ± 1.00	3.24 ± 1.06	3.26 ± 1.11	7.877	0.446	0.699	0.228	3.965	0.138	0.413
I feel more insecure and cautious when handling a similar case.	3.61 ± 0.96	3.61 ± 0.70	3.62 ± 1.23	18.364	***<0.05****	0.127	0.348	0.472	0.790	0.892
I immediately try to change protocols or procedures regarding similar cases.	3.66 ± 1.02	3.45 ± 0.94	3.48 ± 1.07	4.266	0.832	0.898	0.168	1.599	0.449	0.699
I actively seek ways to improve my skills and knowledge to prevent it from happening again.	4.43 ± 0.77	4.27 ± 0.72	4.14 ± 0.78	8.422	0.209	0.495	0.235	5.199	0.074	0.294
I acknowledge that mistakes can happen and do not let it affect my confidence significantly.	3.03 ± 1.01	3.15 ± 1.06	2.64 ± 1.19	14.752	0.064	0.271	0.312	4.563	0.102	0.369
I remain confident in my knowledge and abilities despite the mistake.	3.29 ± 1.05	3.21 ± 0.99	2.83 ± 1.17	8.875	0.353	0.613	0.242	3.985	0.136	0.413

An asterisk (*) denotes a statistically significant association (p ≤ 0.05).
